# Potential increase in coastal wetland vulnerability to sea-level rise suggested by considering hydrodynamic attenuation effects

**DOI:** 10.1038/ncomms16094

**Published:** 2017-07-13

**Authors:** José F. Rodríguez, Patricia M. Saco, Steven Sandi, Neil Saintilan, Gerardo Riccardi

**Affiliations:** 1 School of Engineering and Centre for Water Security and Environmental Sustainability, The University of Newcastle, Callaghan 2308, Australia; 2 Department of Environmental Sciences, Macquarie University, North Ryde 2109, Australia; 3 Department of Hydraulics and Research Council of National University of Rosario (CIUNR), Rosario 2000, Argentina

## Abstract

The future of coastal wetlands and their ecological value depend on their capacity to adapt to the interacting effects of human impacts and sea-level rise. Even though extensive wetland loss due to submergence is a possible scenario, its magnitude is highly uncertain due to limited understanding of hydrodynamic and bio-geomorphic interactions over time. In particular, the effect of man-made drainage modifications on hydrodynamic attenuation and consequent wetland evolution is poorly understood. Predictions are further complicated by the presence of a number of vegetation types that change over time and also contribute to flow attenuation. Here, we show that flow attenuation affects wetland vegetation by modifying its wetting-drying regime and inundation depth, increasing its vulnerability to sea-level rise. Our simulations for an Australian subtropical wetland predict much faster wetland loss than commonly used models that do not consider flow attenuation.

## Abstract (web summary)

Predictions of coastal wetland response to sea-level rise often neglect attenuation effects due to vegetation and infrastructure. Here, the authors show that including attenuation effects improves prediction of wetland evolution and suggests increases in wetland vulnerability to sea-level rise.

Coastal wetlands are among the most productive ecosystems in the world^1^, providing habitat for fish and birds and supporting the productivity of adjacent coastal waters by exporting nutrients^2^. They are particularly vulnerable to sea-level rise due to their location in low lying areas, as shown by a number of studies that have predicted the submergence of 20–78% of worldwide coastal wetland extent by the end of the century^3^,^4^,^5^,^6^. However, these early estimates of sea-level rise impact on wetlands are highly uncertain due to limited understanding of hydrodynamic and bio-geomorphic interactions over time, in particular in areas with high anthropogenic intervention. In fact, recent research has identified the need to incorporate vegetation dynamics processes^7^,^8^ and hydrodynamic effects^9^,^10^,^11^ to achieve more realistic predictions of wetlands response to sea-level rise.

Models of coastal wetland vegetation response to sea-level rise are extensively used in landscape simulation studies^12^,^13^,^14^,^15^, coastal management plans^16^,^17^,^18^ and storm surge predictions^19^,^20^. These models share a common feature: they predict vegetation distribution as a function of the prevailing hydrodynamic conditions. Water depth and duration of inundation have been shown to exert a primary control on vegetation establishment and survival in freshwater^21^,^22^, saltmarsh^23^,^24^ and mangrove^25^,^26^ wetland ecosystems, except in situations with important wave exposure^27^. Different hydrodynamic variables have been used in models to describe vegetation response to water depth and inundation time, including depth below mean high tide^24^, tidal range^12^, hydroperiod^12^,^13^,^14^ and elevation with respect to the tidal frame^28^. One limitation of many of these models is an insufficient detail in the hydrodynamic simulation of flooding processes and attenuation. Commonly used bathtub models assume that the water levels at a given time are the same over the entire wetland^14^,^15^,^16^ thereby neglecting flow attenuation mechanisms altogether. Other models consider hydrodynamic attenuation effects due to vegetative resistance but with a main focus on the channel network^13^,^14^,^29^ and not on the tidal flats, where vegetation establishment occurs. Even in the situations in which some level of attenuation on the tidal flats has been considered^30^,^31^,^32^,^33^,^34^, important modifications to the duration of inundation and its effects on vegetation have not been taken into account. Attenuation reduces wave height and maximum inundation extent but increases ponding time, so it affects both water depths and inundation time and therefore vegetation establishment and survival. In addition to vegetative resistance effects, flood attenuation is increased by man-made flow restrictions like levies, culverts and bridges. These anthropogenic modifications to the tidal regime are already important in many coastal areas, and may become more widespread in the future due to development pressures and flood control measures. The combined effects of vegetation resistance and man-made flow restrictions on wetland evolution under sea-level rise conditions are not considered in any of the existing models.

Here, we use a simulation approach that overcomes these limitations by coupling a detailed hydrodynamic model that fully incorporates attenuation effects with vegetation rules based on preference to hydrodynamic conditions (see Methods section). Spatially distributed hydrodynamic information on water levels is used to compute inundation depths and hydroperiods required by the vegetation for establishment/survival. The rules allow us to predict vegetation distribution using plant-specific information on tolerable water depth and hydroperiod conditions for vegetation establishment and survival during the largest (that is, spring) tides. We first use our approach to model flow attenuation and vegetation distribution on a representative tidal flat with mangroves and saltmarsh. This simple setting is used to investigate the effect of vegetation attenuation in isolation. We then examine a more complex scenario across a tidal wetland with man-made drainage modifications. The second scenario is used to: (1) capture the additional effects of attenuation by man-made hydraulic structures and (2) project the effects of future sea-level rise on wetland evolution. We find that incorporating hydrodynamic attenuation in our approach produces substantial changes in the predictions of wetland evolution compared to those obtained from bathtub approaches, including a much faster rate of wetland loss. Attenuation effects can play an important role in long-term wetland evolution, and must be considered in order to produce accurate predictions of climate change impacts.

## Results

### Flow attenuation due to vegetation on a tidal flat

**Figure 1:**
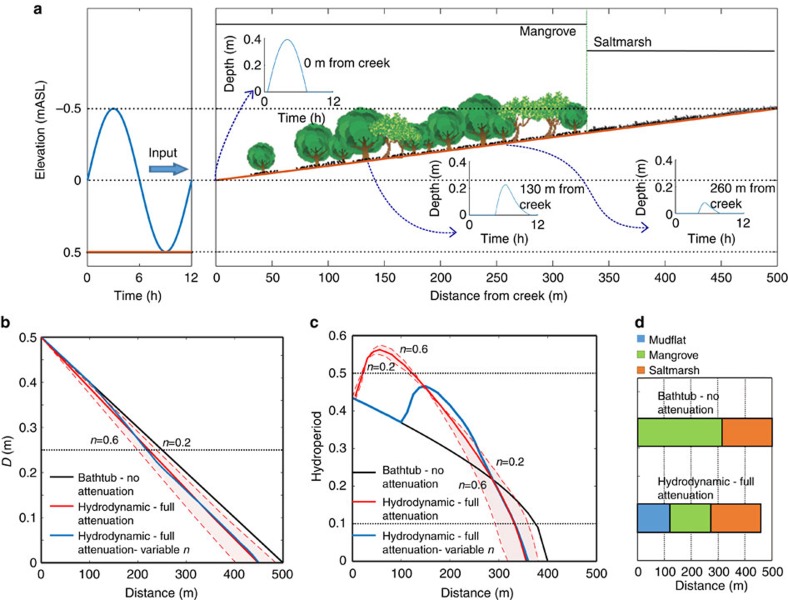
Flow attenuation effects due to vegetation on a subtropical tidal flat. (**a**) Schematic of a typical SE Australian wetland with mudflat-mangrove-saltmarsh sequence illustrating the vegetated tidal flat under a semidiurnal 1-m tidal range signal used for model comparison (vegetation not to scale). Depth hydrographs obtained from the hydrodynamic model at the pneumatophore level at 0, 130 and 260 m from the creek show attenuation of the peak and a longer tail close to the creek (130 m) when the full effects of vegetation attenuation are considered. (**b**,**c**) Changes in *D* and hydroperiod obtained from bathtub and hydrodynamic modelling, showing the attenuation effects of a vegetative flow resistance for a range of Manning’s *n* values from 0.2 to 0.6 (bounds of shaded areas) with a mean of 0.4 (solid red lines), and also the results using a variable *n* value specific for each substrate (0.035, 0.5 and 0.15 for mudflat, mangrove and saltmarsh, respectively) updated based on the full attenuation vegetation distribution in **d** (solid blue line). Changes in hydroperiod are computed at the pneumatophore level, initially set at 14 cm from the ground. (**d**) Vegetation distribution simulated using the bathtub and the hydrodynamic approaches for *n*=0.4. Modelled vegetation responds primarily to preferences to threshold values for *D* (in the case of saltmarsh) and hydroperiod (in the case of mangrove) indicated as dotted horizontal lines in **b**,**c**, respectively. Metres above the Australian height datum (mAHD) in (**a**) stands for metres above the Australian height datum, which approximates mean sea level.

To compare the predictive capability of our current approach with that of previous models that do not properly account for attenuation effects, we first analyse the very simple case of a tidal flat colonized by mangrove and saltmarsh. We compute water levels on the flat as a result of the tidal discharges of a creek using both the bathtub methodology and our approach that, in this case, includes flow attenuation induced by vegetation resistance only (see Methods section). Vegetation resistance is accounted for through a Manning’s resistance coefficient *n* of 0.4±0.2, which covers the range usually reported for the vegetation considered^35^,^36^,^37^. Since the distribution of vegetation is not known *a-priori*, a constant value of Manning’s *n* is initially assumed over the entire mudflat. The conditions used for the model set-up correspond to a typical coastal wetland of SE Australia^38^,^39^ (Fig. 1a), with a slope of 0.001 m m^–1^, and a sinusoidal semi-diurnal spring tide of 1-m tidal range. Vegetation species on the tidal flat include grey mangrove *Avicennia marina* and *Sporobolus virginicus*–*Sarcocornia quinqueflora* mixed saltmarsh.

We use the same vegetation establishment/survival rules based on depth below mean high tide *D* and spring-tide hydroperiod *H* in the two approaches. However, these two hydraulic variables affect mangrove and saltmarsh differently, as explained in the Methods section. Values of hydroperiod are computed along the tidal flat (Fig. 1c) at an offset reference elevation of 14 cm above the tidal flat surface. The offset allows for the computation of the hydroperiod at the top of mangrove pneumatophores, which are assumed to be 14-cm high based on observations (Table 1). The pneumatophore height is a more representative position than the soil surface to assess mangrove survival as it is the part of the plant that is sensitive to inundation.

The comparison of hydraulic results from the two models depicted in Fig. 1b,c show that attenuation effects have a considerable impact on the simulated values of both *D* and hydroperiod along the tidal flat. Inundation extent and maximum depth (represented by *D*) are reduced by up to 20% by attenuation, but the effects on hydroperiod are not as straightforward. Close to the creek there is an increase in hydroperiod, meaning that flow attenuation increases the duration of pneumatophore submergence conditions. This effect decreases rapidly further away from the creek. Figure 1a shows depth hydrographs computed using the hydrodynamic model at distances of 0, 130 and 260 m from the tidal creek and clearly illustrates that flow attenuation reduces the peaks and generates longer tails in the proximity of the creek. The reduced peaks impact the values of *D* while the longer tails result in longer inundation times and thus larger hydroperiods close to the creek.

Using the values of *D* and hydroperiod from the two approaches and the rules for vegetation establishment, we can define a vegetation profile for each case (Fig. 1d). The bathtub approach predicts that mangrove covers 60% of the tidal flat and saltmarsh the other 40% at the highest elevations. When attenuation is considered using the initial uniform resistance value of *n*=0.4 the resulting vegetation mosaic includes a mudflat area of 25%. This area is inundated for too long for mangrove to establish, resulting in a reduction of mangrove coverage to 35% but not affecting saltmarsh extent, which remains at 40%. After the initial simulation, we can assign to each substrate of the vegetation profile (including mudflat) a specific value of *n,* and perform a new run of the hydrodynamic model. Using the vegetation profile obtained with the hydrodynamic approach and substrate-specific calibrated *n* values of 0.035, 0.5 and 0.15 for mudflat, mangrove and saltmarsh, respectively (see Methods section), we then update *D* and the hydroperiod (blue lines in Fig. 1b,c). These new values of *D* and hydroperiod will determine a new vegetation profile different from the original one and iterations and adjustments to the *n* values will be required. In particular, the area classified as mudflat must be considered a transitional area as it can still support a few mangroves. A sparse coverage will generate a local value of resistance considerably lower than *n*=0.5 but larger than the mudflat *n* value of 0.035, which will result in a reduction of the hydroperiods in the transitional area towards values that are tolerable by mangroves. The prediction of a sparse mangrove coverage at the seaward edge of the mangrove forest is consistent with observed wetland profiles for mild slopes.

A common simplification in many wetland models^12^,^13^,^14^ is the use of *D* as a proxy for *H*, which implicitly assumes a unique relation between these two variables. For situations in which attenuation effects are important the use of such simplification is unrealistic because *D* and *H* respond differently to changes in flow conditions. For example, using a model in which *H* is linearly related to *D*^30^ produces considerable errors in the estimation of *H* and thus a vegetation distribution that is very similar to the bathtub distribution (see Methods section for model details and Supplementary Fig. 1 for results).

### Flow attenuation due to vegetation and man-made structures

**Figure 2:**
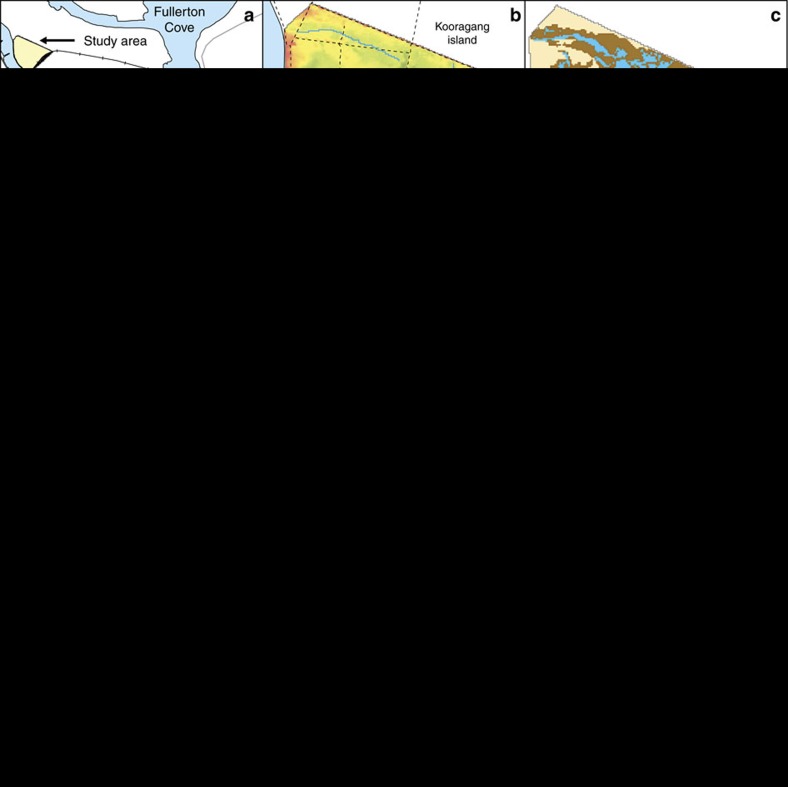
Effect of flow attenuation due to vegetation and infrastructure on a subtropical coastal wetland. (**a**) Map of the Hunter estuary showing, in yellow, the location of the wetland used in this study. (**b**) Tidal flow in the study site is restricted by high embankments over most of its perimeter with two inlets from the Hunter River and a number of internal channels and culverts. Topographic elevations range from −1 to 3.5 mAHD, but most of the tidal flats are within 0–1 mAHD (**c**) The 2004 survey of the site show mangrove establishment in the lower wetland and saltmarsh at higher elevations, with considerable areas of mudflat and tidal pools. (**d**,**e**) When no flow attenuation is considered the annual values of hydroperiod and *D* during spring tides are only governed by the tidal cycle and the topography. (**f**) The predicted vegetation distribution based on unattenuated values of hydroperiod and *D* grossly overestimates the extent of mangrove when compared to the distribution observed in **c**. (**g**,**h**) When flow attenuation is considered the annual values of hydroperiod and *D* during spring tides show more areas that are permanently inundated (0.8<hydroperiod<1) and have shallow depths (0<*D*<0.3) in the northern half of the wetland than in the previous case. (**i**) The predicted vegetation distribution based on values of hydroperiod and *D* computed with attenuation (**g**,**h**) results in the northern part of the wetland dominated by saltmarsh and tidal pools, and mangrove circumscribed to the southern part of the wetland in agreement with the distribution observed in **c**. mAHD in (**b**) stands for metres above the Australian height datum, which approximates mean sea level.

We now consider the additional attenuation effect of existing man-made flow control structures such as roads, embankments and culverts that affect many coastal wetlands around the world. We illustrate the potential effects of attenuation using as a case study a subtropical coastal wetland located in the Hunter estuary of SE Australia, which is presented in Fig. 2a,b. The wetland includes 10 active culverts and bridges over an area of 1.2 km^2^. This level of concentration of flow control structures follows the regional trend in this part of Australia^40^, but similar values have also been reported for saltmarsh in New England and the mid-Atlantic US coast^41^. Inflows from the South Arm of the Hunter River enter this wetland by two control structures: a pipe culvert at Wader Creek inlet and a bridge at Fish Fry Creek. Several internal culverts control the flow of water within the wetland and the outer perimeter is delimited by high embankments with no connection to the rest of the island. This wetland includes most of the estuarine habitats present in the Hunter estuary and typical of SE Australian wetlands, including mudflat, tidal pool, *A. marina* mangrove and *S. virginicus*–*S. quinqueflora* mixed saltmarsh. Figure 2c shows the distribution of vegetation surveyed in 2004.

As we did in the case of the tidal flat, we compare the vegetation distribution resulting from the bathtub and the full attenuation approaches when applied to the 2004 conditions. The implementation of the hydrodynamic model for the full attenuation approach required discretization of the domain into a dense grid (10 × 10 m cells), delimitation of creeks and tidal flats and inclusion/set-up of 10 control structures. Standard discharge coefficients were adopted for the control structures and spatially varying resistance coefficients for vegetated, unvegetated and creek areas were obtained from calibration to best reproduce existing measured hydrodynamic data (see Methods section and Supplementary Tables 1–3). For the two approaches, we compute values of *D* and the hydroperiod during spring tides using as boundary condition at the wetland inlet tidal records from a nearby gauging station with a 15-min resolution (Fig. 2d,e,g,h). The vegetation rules (see Methods section) are the same as previously used for the tidal flat simulations, although we introduce an additional secondary restriction for saltmarsh establishment of hydroperiod <0.8 to represent tidal pools in areas that have very shallow depth but are almost permanently inundated. The comparison of model results for current wetland conditions with observations (Fig. 2c) shows that the full attenuation approach (Fig. 2i) captures the general vegetation distribution of the site, whereas the bathtub approach (Fig. 2f) grossly over-predicts mangrove cover.

### Flow attenuation and sea-level rise

To assess sea-level rise effects, we run long-term simulations with both the bathtub and the full hydrodynamic approaches until the wetland becomes submerged. Over such time scales it is necessary to incorporate the effect of wetland soil surface elevation change, due to the capability of vegetation communities to build up their own soil by trapping sediments and by accumulating organic matter^7^,^42^. Surface Elevation Table (SET)^42^ measurements at our study site (Table 1) indicate that the soil surface elevation over the period 2002–2012 has increased at a rate of 1.39 mm yr^−1^ in saltmarsh areas and 2.23 mm yr^−1^ in mangrove areas^43^, which is in line with other SE Australia’s coastal wetlands with similar low values of sediment input^28^. We use these data to predict future surface elevation change following two different approaches (see Methods section). The first approach assumes a constant rate of soil surface elevation change identical to the 2002–2012 data, based on the fact that vegetation over that period was already responding to sea-level rise^23^. The second approach uses the SET data to calibrate a variable rate of soil surface elevation formulation that is explicitly affected by sea-level rise due to eco-geomorphic feedbacks, in line with previous research^7^,^12^.

We consider a sea-level rise rate during this century that corresponds to the IPCC AR5 PCP8.5 scenario^44^ and we use two different simulation approaches. We apply a constant rate of 8 mm yr^−1^ for the simulations with the constant rate of surface elevation change, and a variable rate gradually accelerating from 4 mm yr^−1^ in 2000 up to 11 mm yr^−1^ in 2100 for the variable rate of surface elevation change. Input water levels consist of the tidal signal used in the one-year simulations (Fig. 2) and considering a cumulative increase given by the corresponding sea-level rise. We run the hydrodynamic model continuously for each year, but we use a 20-year time step to update vegetation distribution, to raise soil surface elevations in the vegetated areas and to update roughness coefficients *n* in areas of vegetation change. As with all other long-term wetland evolution studies^12^,^13^,^14^,^15^, this 20-year time step is a compromise between computational time and process description and is compatible with the slow dynamics of the system.

**Figure 3:**
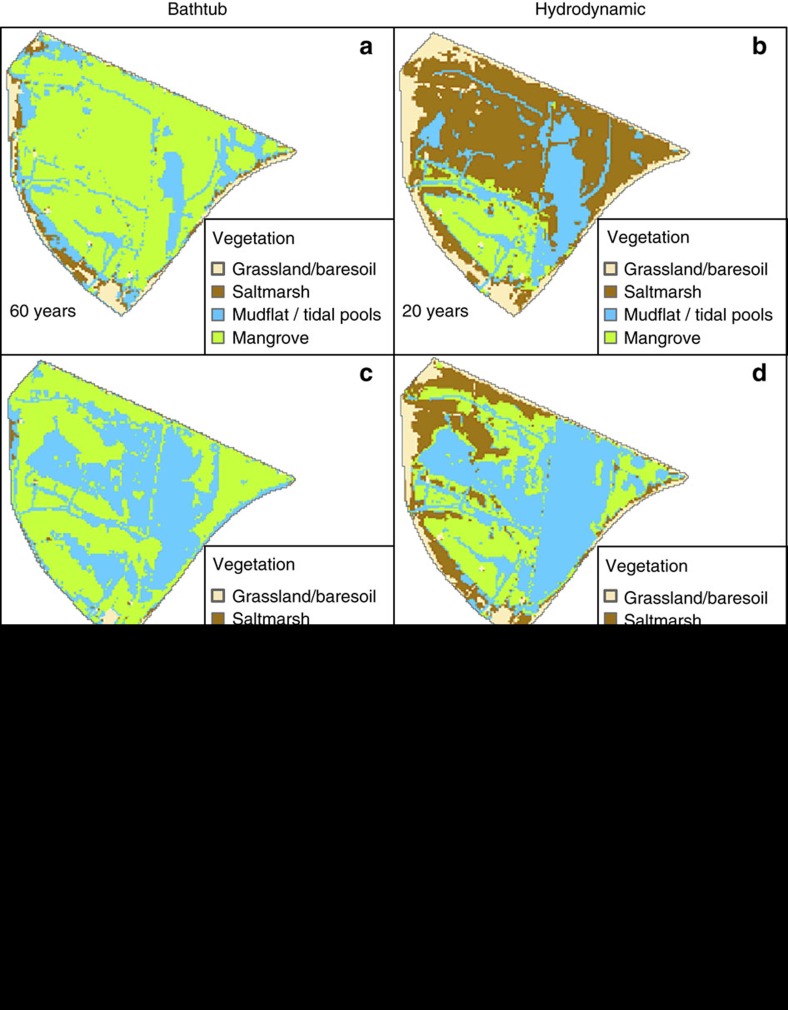
Wetland vegetation changes due to constant sea-level rise and constant soil surface elevation change. (**a**,**b**) 30–40% of vegetated area loss is predicted after 60 years in the bathtub model but considerably sooner (20 years) in the attenuated case. During this initial phase losses are partially compensated by colonization of higher buffer zones. (**c**,**d**) 50–55% of vegetation loss occurs after 80 and 40 years according to the bathtub and hydrodynamic model, respectively. Faster losses in this phase are associated with the disappearance of buffer zones. (**e**,**f**) 80–85% of vegetation loss at 120 years (bathtub model) and 80 years (hydrodynamic model) in this last phase indicates a slower rate of inundation related to the steeper relief of the higher areas of the wetland in the periphery. For complete results see Supplementary Figs 2 and 3.

Figure 3 shows snapshots of the bathtub and hydrodynamic model simulations at different times for the first approach with constant rates of both sea-level rise and soil surface elevation change. The snapshots have been selected from the full set of model outputs (Supplementary Figs 2 and 3) to illustrate that similar vegetated area loss percentage occurs at different times for the two models. As mentioned before the bathtub model over-predicts the initial distribution of mangroves (Fig. 2f), and without flow attenuation effects the changes in *D* and hydroperiod over time simply reflect the net elevation difference between sea-level rise and surface elevation gain^28^ (Fig. 3a,c,d). The transition of mangrove to mudflats and tidal pools occurs in the lowest elevation zones of the wetland due to increasing hydroperiods (Supplementary Fig. 2). Almost total wetland loss (80%) occurs after ∼120 years, which is consistent with previous predictions for this area^28^. When attenuation effects are considered the long-term vegetation distribution evolves faster following the pattern shown in Fig. 3b,d,f. Attenuation results in a complex nonlinear relation between hydroperiod and *D* (Supplementary Fig. 3), as opposed to the approximate linear link between these variables when a simpler flow dynamics is considered^12^,^13^,^14^,^15^,^16^,^17^,^18^,^28^. As a result of sea-level rise, saltmarsh is initially displaced by mangrove and by the increasing depths (Fig. 3b,d) while mangrove is adversely affected by the increasing hydroperiods later on (Fig. 3f). During the first 20 years there is a minor migration to higher ground in both mangrove and saltmarsh communities as a result of increasing depths and hydroperiods (Fig. 3b), which compensates vegetation losses due to the increase in the size of tidal pools. The next two snapshots at 40 and 80 years show more pronounced vegetation losses due to an increase in permanently inundated areas that have large hydroperiods and depths (Supplementary Fig. 3). The final distribution of vegetation (Fig. 3f) consists of mangroves fringing a large central tidal pool with remnant saltmarsh in the periphery, which agrees with a pattern increasingly observed in subtropical wetlands^45^ but not previously predicted in models. Figure 3 clearly shows a much faster loss of wetland vegetation when attenuation effects are considered. Our model results predict that the vegetation loss rate is almost twice the value predicted by previous models that do not consider flow attenuation.

**Figure 4:**
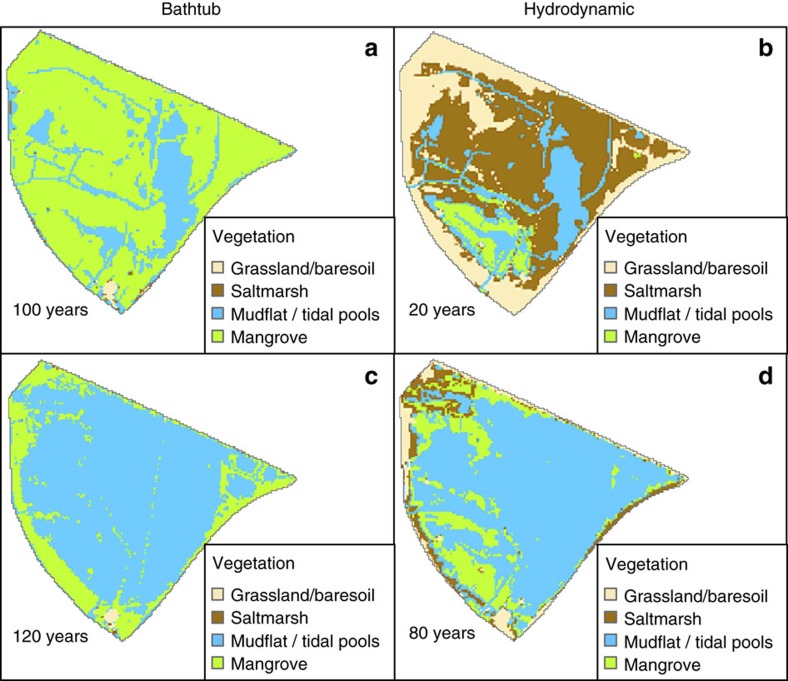
Wet land vegetation changes due to variable sea-level rise and variable soil surface elevation change with low suspended sediment. (**a**,**b**) 25–45% of vegetated area loss is predicted after 100 years in the bathtub model but considerably sooner (20 years) in the attenuated case. During this initial phase changes are slow due to low sea-level rise rates and also because losses are partially compensated by colonization of higher buffer zones. (**c**,**d**) After the initial period of slow change due to low rates of sea-level rise, wetland loss trajectory is similar to the results using constant rates of sea-level rise and a constant rate of surface elevation gain achieving 75–80% of vegetation loss at 120 years (bathtub model) and 80 years (hydrodynamic model). For complete results see Supplementary Figs 4 and 5.

Nonlinear eco-geomorphic feedbacks can produce increases in the rates of surface elevation gain above the historic rates as sea-level rise rate increases^7^,^12^. If these changes in surface elevation are important, they may compensate for the higher loss rate predicted by the effects of flow attenuation. In Fig. 4, we compare bathtub and hydrodynamic predictions of wetland evolution using a variable rate of sea-level rise and relaxing the assumption of a constant soil surface elevation change rate by including nonlinear eco-geomorphic feedbacks (see Methods section). The selected snapshots of the complete simulations of Supplementary Figs 4 and 5 show slower initial vegetation dynamics compared to the results of Fig. 3 due to lower rates of sea-level rise for the first few years (Fig. 4a–d). However, for longer simulation times we see a remarkable similarity to the previous results given by the first approach that considered constant historic rates (Figs 3e,f and 4c,d). Further analysis on the evolution of the values of *D* for attenuated conditions (Supplementary Fig. 5) on vegetated areas and the associated variable surface elevation dynamics (equations (7) and (8)), reveals that the spatially averaged surface elevation gains of both saltmarsh and mangrove converged to the historic values of the first approach. Surface elevation change gains rates for the bathtub simulations were about twice the historic values, but still not enough to prevent extended wetland submergence as shown by Supplementary Fig. 4.

The initial period of slow vegetation changes lasts 80 years for the bathtub model, followed by a sudden transition to mudflat after 100 years (Supplementary Fig. 4). The hydrodynamic model predicts a more gradual vegetation loss, with an initial slow change period of 40 years followed by a period of accelerated losses coinciding with the accelerated sea-level rise (Supplementary Fig. 5). As with the constant surface elevation change rate model, flow attenuation increases the rate of vegetation loss by a factor of two.

**Figure 5:**
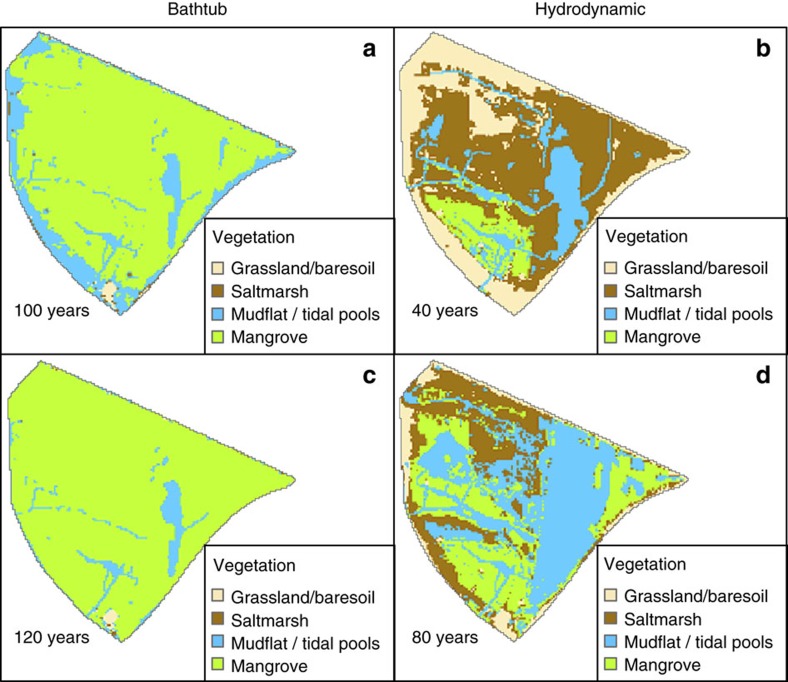
Wetland vegetation changes due to variable sea-level rise and variable soil surface elevation change with high suspended sediment. (**a**,**c**) With the increased sediment availability bathtub model results indicate that the wetland withstands sea-level rise, with no loss of vegetation predicted. (**b**,**d**) The hydrodynamic model results still predicts considerable wetland loss, with about 70% of vegetated area loss after 80 years. However, due to increase sediment availability the rate of vegetation loss is half the rate of the low suspended sediment simulations of Fig. 4. For complete results see Supplementary Figs 6 and 7.

The predictions of Fig. 4 incorporate a variable rate of soil surface elevation change that strongly depends on the availability of suspended sediment *C* (equation (7) in Methods section). The results shown in Fig. 4 are based on constant *C* values of 15 g m^−3^ for saltmarsh and 22 g m^−3^ for mangrove for the entire simulation period based on measurements for our wetland^46^. While this low value is consistent with the low suspended sediment concentrations typical of the Hunter estuary and other SE Australia sites^28^, it is of interest to investigate how wetland vegetation dynamics can be affected by flow attenuation in a situation with a higher level of sediment availability. In Fig. 5, we compare bathtub and hydrodynamic predictions of wetland evolution using *C* values of 45 g m^−3^ for saltmarsh and 66 g m^−3^ for mangrove under the same accelerating sea-level rise trajectory used in the simulations of Fig. 4. According to previous research^28^ this higher level of sediment availability can potentially result in surface elevation gains that compensate sea-level rise, thus preventing ultimate wetland drowning.

With the increased sediment availability, the bathtub model predicts a vegetation cover that remains stable with no appreciable vegetation loss for the entire simulation period (Fig. 5a,c and Supplementary Fig. 6). However, predictions using the hydrodynamic model still produce appreciable levels of wetland loss (Fig. 5b,d and Supplementary Fig. 7). The rate of vegetation loss is about half of the rate predicted by the hydrodynamic model under low sediment availability conditions as can be seen when comparing Supplementary Figs 5 and 7. The initial period of slow changes extends to 60 years, which is larger than the slow change period of the simulations with low sediment availability.

Analysis of the surface elevation dynamics for the entire wetland indicate that in the bathtub simulations elevation gain rates reach values six times larger than historic rates and three times the rates of the low sediment load situation. Elevation gain rates in the case of attenuated conditions were much lower, about twice the historic rates and the low sediment load rates.

These last simulations with a variable accretion rate consider that the sediment concentrations for saltmarsh and mangrove are different from each other but spatially constant within each vegetation type. Additional simulations carried out using a spatially variable value of *C* that captures the observed decay of concentration as a function of distance to the main inlet at Fish Fry Creek (Supplementary Fig. 12) display the same general trends as the constant *C* simulations (see Methods section for model details and Supplementary Figs 8–11 for results).

## Discussion

Our study suggests a significant underestimation of sea-level rise impacts predicted by commonly used models on coastal wetlands affected by man-made flow restrictions. The lack of an appropriate hydrodynamic flow description, common in the models, is a key reason for the underestimation because the underpinning bathtub approach used to compute water levels within the wetland cannot simulate flow attenuation effects due to vegetation, culverts, levies and bridges that distort the tidal wave. Attenuated flow produces less tolerable conditions for wetland vegetation (Fig. 1).

In our new approach, we capture the effect of those attenuated flow conditions on water depth and duration of inundation, which can then be linked to physiological requirements of the vegetation. We model these vegetation requirements based on well-documented observations showing that saltmarsh cannot survive if totally submerged^24^ and that mangrove roots cannot withstand submergence for more than half of the duration of the tidal cycle^26^. Application of our hydrodynamic-based model to predict the observed vegetation distribution in an Australian subtropical wetland with man-made flow restrictions produced remarkably good results. In contrast, results obtained using the traditional bathtub approach could not reproduce the observed distribution (Fig. 2).

Our long-term projections of wetland loss rate induced by sea-level rise (Fig. 3) including historic surface elevation change gains almost doubled when compared to the rate given by our bathtub-based projections that do not account for flow attenuation. The higher loss rate was observed even for the case in which we incorporated sediment-dependent bio-geomorphic feedbacks that have been shown to potentially allow wetlands to adjust to changes in sea-level rise by increasing their surface elevation gain rate^7^,^12^. With a low sediment input typical of eastern Australian wetlands, the feedbacks were unable to produce substantial increases in surface elevation gain to prevent wetland drowning (Fig. 4). Increasing the sediment input to levels that can potentially result in wetland surviving sea-level rise according to bathtub-based predictions^28^ did produce higher surface elevation gain rates; however, these rates were not enough to prevent substantial drowning (Fig. 5). The limited capacity of the nonlinear eco-geomorphic feedbacks to keep up with sea-level rise under attenuated conditions was due to the low depths induced by attenuation, which were less than required to produce enough surface elevation gains.

The main reason for the higher loss rate is that in most of the wetland, hydroperiods are larger when attenuation is considered, and in addition their rate of increase as a result of sea-level rise is higher. To illustrate the magnitude of this effect, we can focus on a point in the northern part of the wetland (point WPW in Fig. 2) where flow attenuation is more pronounced and analyse changes in hydroperiod in the simpler case of constant rates of sea-level rise and surface elevation change. Sea-level rise impact predictions (Supplementary Fig. 3) for WPW resulted in an average rate of increase of hydroperiod of 0.0075, yr^−1^ during the first 80 years of simulation, which was three times faster than the rate of 0.0025, yr^−1^ obtained with a bathtub model (Supplementary Fig. 2). During the same period the rate of increase of inundation depths *D* at WPW did not show substantial effects of attenuation, as both our model and the bathtub approach predicted the same rate of increase. The rate of increase corresponded to the difference between sea-level rise rate and soil surface elevation change at the point.

On the basis of our results for saltmarshes and mangroves we find that, by considering the effects of flow attenuation, predicted rates of wetland loss are higher than those forecasted with bathtub approaches. These effects will be larger in wetlands with high density of flow restrictions. To realistically represent the evolution of our study wetland, our results include the combined effects of man-made flow restrictions and vegetation resistance. Future work is needed to further assess the individual effects of each attenuation mechanism. This is particularly important as man-made alterations to flow regime affect flows into wetlands, and therefore sea-level rise impacts on heavily developed coastal areas of the world, like eastern Australia^40^, eastern US^41^, eastern China^47^ and Western Europe^48^. The geographical extent of man-made structures can be expected to increase as population increases. Under these conditions, the successful management of future wetlands for coastal retreat and realignment strategies^49^ will require careful consideration of the effects of floodplain structures on hydroperiod and inundation depth affecting the growth and survival of wetland plants.

## Methods

### Modelling approach for wetland evolution

We model the distribution of wetland vegetation by first determining the prevailing spatially distributed hydrodynamic conditions and then applying simple vegetation rules based on hydrodynamic preferences of saltmarsh and mangrove. The prevailing hydrodynamic conditions are obtained by integrating values of depth below mean high tide *D* and spring-tide hydroperiod *H* during a year of tidal inflows into the wetland based on gauge data from 2004 (Table 1). For the long term simulations of wetland evolution under sea-level rise scenarios, we consider annual increments of the tidal inflows incorporating both the rate of sea-level rise and the soil surface elevation changes in saltmarsh and mangrove. Due to the slow wetland dynamics, we update vegetation distribution and the associated changes in soil surface elevations and Manning’s *n* resistance values every 20 years. In all cases, we compare vegetation distributions obtained using values of *D* and hydroperiod from two approaches: the simplistic bathtub type flow simulations and the full hydrodynamic simulations that captures the effects of flow attenuation.

### Conditions for vegetation establishment/survival

**Figure 6:**
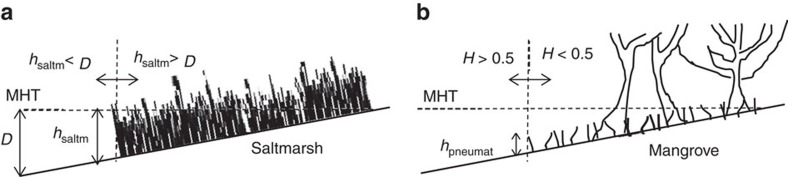
Main conditions for vegetation establishment/survival. (**a**) Saltmarsh establishment is limited by inundation depths *D* larger than its height *h*_saltm_ due to hypoxia. (**b**) Mangrove establishment, on the other hand, is limited by its pneumatophores being able to access oxygen at least half of the time, which is given by a hydroperiod *H* smaller than 0.5 at the pneumatophore height *h*_pneumat_. MHT stands for Mean High Tide.

Vegetation is considered to depend on spring-tide flow conditions, when inundation is more pronounced and therefore critical for vegetation survival. For these conditions, the relevant hydraulic variables are depth below mean high tide *D* and spring-tide hydroperiod. Since hypoxia limits establishment for saltmarsh^20^, we set a tolerance limit of *D*<25 cm for establishment/survival of the saltmarsh of our typical SE Australia site that has a height of 25 cm (Fig. 6a). The relationship between vegetation height and inundation depth is a simple yet strong predictor of saltmarsh survival, as shown by our observations (Table 1) and previous studies in marshes of South Carolina^24^,^50^. We also introduce a secondary restriction of hydroperiod <0.8 for the establishment of saltmarsh in areas almost permanently inundated in order to represent tidal pools.

Mangrove is considered to be sensitive to spring-tide hydroperiod, and we adopt a suitable range 0.1<*H*<0.5 to model the effect of limited oxygen availability and accumulation of phytotoxins in soils^26^ (Fig. 6b). Again, these values are selected based on our *A. marina* data (Table 1) but are consistent with data from Northern Australia for *Rhizophora stylosa*^26^, a species of mangrove that occupies a similar position in the tidal frame to *A. marina*. We also impose a secondary constraint of a minimum inundation level of 0.2 m to avoid shallow areas that are too saline for *A. marina* propagules to survive^51^. If hydrodynamic conditions allow for both vegetation species to establish, it is assumed that mangrove will outcompete saltmarsh in this type of wetland^52^.

### Calculation of *D* and *H* with the bathtub flow model

The bathtub model assumes that at any given time water levels are the same over the entire wetland. Consequently, the *D* values can be obtained directly by subtracting the topographic elevation from the mean high tide level. In the case of the tidal flat of Fig. 1, *D* can be computed as


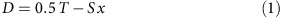


where *T* is the 1-m spring tidal range*, S* is the slope of the tidal flat (0.001 m m^−1^ in our case) and *x* is the horizontal distance from the creek. Equation (1) corresponds to the line labelled bathtub-no attenuation in Fig. 1b. In the application to the entire SE Australia wetland site of Fig. 2, *D* is obtained using the more complex topography and an average of the high tide levels of all spring-tide periods in the tidal record corresponding to the simulation (Fig. 2e).

We compute hydroperiod as the ratio between the time interval during which a given point is submerged to the total duration of the period of reference. The spring-tide hydroperiod *H,* is calculated by averaging the proportion of inundation time of all spring tides. Over the tidal flat, we consider a sinusoidal spring tide of the general form *z*=0.5 *T* cos (2*π t*/*λ*) with *z*=water surface elevation*, t*=time and *λ*=wave period. For this sinusoidal wave the expression for *H* can be obtained by noting that *H*=2*t*/*λ* when *z*=0.5 *T*−*D*, that is,


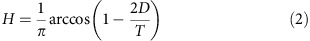


Equation (2) was used to calculate the bathtub-no attenuation curve in Fig. 1c. In the case of the entire wetland, numerical integration of wet time intervals during spring tides is required to compute the hydroperiods shown in Fig. 2d.

### Calculation of *D* and *H* with the hydrodynamic flow model

The spatial distributions of *D* and *H* are obtained from the continuous values of water levels provided by the hydrodynamic model. Over spring-tide periods modelled water levels are used to compute the mean high tide level required for the *D* values, and also to keep track of the wet and dry periods required for the *H* values. The model uses a two-dimensional finite difference method to solve the shallow water equations by means of a cells scheme^53^, and we apply the same model to both the tidal flat and the entire wetland. We represent the tidal flat as a 500-m long platform with the tidal creek water elevations as a boundary condition. The platform is discretised into 12 × 12 m square cells and is very wide so that the effects of lateral model boundaries are minimal. For the entire wetland model we discretise the domain into 10 × 10 m square cells resulting in a total of 13,543 cells.

At each time step, the hydrodynamic model first solves mass conservation to provide water surface elevations:


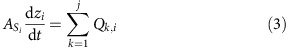


where *A*_S*i*_ and *z*_*i*_ are surface wetted area and water surface elevation at cell *i*, respectively, and *Q*_*k,i*_ are the discharges between cell *i* and its *j* neighbouring cells. The discharges between cells are computed next using the momentum or energy equation based on the water surface gradients and restrictions to flow. The discharge equations are different depending on the type of connection between cells. For example, if two cells are on the vegetated tidal flat, the discharge between them is be calculated using Manning’s *n* as:


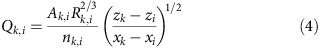


where *A*_*k,i*_ *R*_*k,i*_ *n*_*k,i*_ are respectively the cross-sectional values of area, wetted perimeter and Manning roughness computed as an average of the values at cells *k* and *i*, and *x*_*k*_*−x*_*i*_ is the distance between cells. If one of the cells is a control structure, for example, a culvert at cell *i*, then the discharge between cells is computed as:


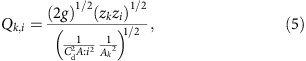


in which *A*_*i*_ and *A*_*k*_ are respectively the cross-sectional areas at the *i* and *k* cells and *C*_d_ is a standard discharge coefficient for the culvert at cell *i*. For the wetland model, a value of *C*_d_=0.8 was used for all culverts as they have similar discharge characteristics.

To handle the wetting and drying process efficiently, cells are classified into channel and tidal flat categories. Channel cells have a trapezoidal cross-sectional channel in the direction of the flow, while tidal flat cells have two small virtual trapezoidal channels in the two possible flow directions that intersect in the middle of the cell. The size, shape and slope of the virtual channels have little effect on the overall computations as their depth is only 0.01 m. Any cell with a depth less than 0.01 m is considered a dry cell. These artificial sub-grid features avoid discontinuities in water levels between cells, which are the main problem when tracking the wet-dry front. All cells in the tidal flat model are tidal flat cells, while the wetland model requires the specification of channel and tidal flat cells. The model equations are solved using an implicit finite difference method and a Newton–Raphson algorithm^53^.

The wetland model requires calibrated *n* values to correctly capture the complex flow dynamics. We performed standard calibration using field data by varying the value of *n*. Two different water depth time series were used for calibration and validation. The time series are records obtained using pressure transducers during 2004 and 2005 (Supplementary Table 1) for three locations in the study site: TGB, WPW and Bridge (Fig. 2b). The record from Bridge is located at the Fish Fry Creek inlet and was used as input water level for the model. The water depth series from Bridge was also used as input water level at the Wader Creek inlet with the assumption that changes in the water surface profile along the south arm of the Hunter River can be neglected over such a short distance. The records at the other two locations were used for calculating the performance of the model.

Calibration of the model consisted of obtaining the combination of Manning’s *n* roughness coefficients for areas with different soil coverage that showed the best overall performance. On the basis of measured data (Table 1) a range of *n* values for each land use was tested. Supplementary Table 2 shows the range of Manning roughness coefficients that were tested for each land use and the values from the best combination obtained after calibration. Validation using the calibrated *n* values was performed on an independent time series collected around the time of the King Tide, during which water levels were considerably higher than during calibration.

The best model fit to the field data was selected based on a number of statistical indicators and resulted in values of *n*=0.035 for channel and unvegetated substrate, *n*=0.15 for saltmarsh and *n*=0.5 for mangrove.

The performance of the model was evaluated using four statistical indicators customarily used in hydrological models^54^: the Pearson’s correlation coefficient (*r*), the ratio of the root mean square error to the standard deviation of measured data (RSR), the Nash-Sutcliffe efficiency (NSE), and the per cent bias (PBIAS). Average values of the indicators during calibration and validation of the model are presented in Supplementary Table 3, where it can be seen that the model performance was satisfactory when using as a guide the following recommended values^54^: *r*>0.5, RSR<0.70, −25%<PBIAS<25% and NSE>0.50. The validation series presented values of RSR and NSE that were just inside of the recommended range, but the validation was considered acceptable as the series was quite short and the other two indicators (*r* and PBIAS) showed a very good model performance. Comparable calibration and validation results have been reported for similar wetland environments using a similar distributed hydrodynamic model^33^. Of particular importance for the long-term model operation was the verification of an unbiased prediction (low PBIAS) as systematic over- or under-predictions generate over time unrealistic volumes of water in the many storages of the wetland.

### Simplified flow attenuation formulation

A common simplification in many wetland models is the assumption of a linear relation between *D* and hydroperiod *H*, which justifies the use of *D* (or position in the tidal frame) as a proxy for *H*. The assumption is not valid when important attenuation effects are present as a result of, for example, vegetation resistance. To show the limitations of such approach, we have redone the computations presented in Fig. 1 for the tidal flat using a simplified approach presented in previous work^30^ that combines a hydrodynamic-model calculation for *D* and a simplified computation of hydroperiod as a linear function of *D*.

The simplified model is based on the computation of *D* including flow attenuation (in the form of Manning’s resistance coefficient in our case), which was carried out using the same formulation than the full hydrodynamic model. For *H*, however, instead of integration of wet periods a direct formula was used. The formula can be obtained by linearly approximating the equation that relates *H* and *D* without the consideration of flow attenuation (equation (2)) in the range 0.2<*D/T*<0.8 (ref. 30).


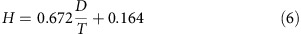


Supplementary Fig. 1 shows the comparison of the simplified model results with the results of the full attenuation model in terms of *D, H* and vegetation distribution. Also in Supplementary Fig. 1, we have included the results of the no attenuation or bathtub approach. The *D* values of the simplified model are computed using a hydrodynamic formulation so they coincide with the full attenuation results (Supplementary Fig. 1a). However, the values of *H* that result from using the simplified model are practically unaffected by attenuation (Supplementary Fig. 1b) and are closer to the bathtub model values. The vegetation distribution that results from combining the simplified model with the vegetation rules used in Fig. 1 shows a similar vegetation coverage than the one obtained using the bathtub approach. This simplified model was only applied to the tidal flat and not to the whole wetland as it did not show any substantial improvement over the model with no flow attenuation.

### Sea-level rise for long-term simulations

We consider a sea-level rise during this century that corresponds to the RCP 8.5 scenario^44^. To test and compare our results with previous regional estimates that use a constant sea-level rise^28^, we use a constant rate of 8 mm yr^−1^. For comparison with other models that consider both a variable sea-level rise rate and a variable rate of soil surface elevation change^7^,^12^, we include an accelerated rate of sea-level rise gradually increasing from 4 mm yr^−1^ in 2000 up to 11 mm yr^−1^ in 2100. We do not consider changes in the amplitude of tides due to sea-level rise and we also neglect any modulation effects of the estuary. This simplification is supported by data from the nearby Hexham Bridge tide gauge for the period 1990–2010 (ref. 55) that shows that the estuary mean water level, mean high tide and mean low tide all rose at the same rate, indicating that tidal amplitude was not affected by sea-level rise. A very similar rate of water level increase was recorded at the closest ocean tide gauge at Tomaree for the same period^43^, indicating a negligible modulating effect of the estuary. The Tomaree gauge is a good indicator of sea-level rise due to its proximity to the site and is part of a network of ocean gauges located outside ports and estuaries that are not influenced by local coastal features.

### Surface elevation change due to eco-geomorphic feedbacks

For testing and comparison of our model results with regional bathtub model prediction over the Indo-Pacific region^28^, we consider a constant rate of change of soil surface elevation of 1.39 and 2.23 mm yr^−1^ for saltmarsh and mangrove, respectively. These rates correspond to a 10-year record (2002–2012) of surface elevation changes derived from SET deployed on site, as detailed in Table 1. The surface elevation change trajectory derived from these data, one of the longest continuous SET measurement records in the world, integrates changes over a period of rising sea-level^43^. Values of constant rate of surface elevation change are combined with the constant rate of sea-level rise of 8 mm yr^−1^ for the long-term simulations.

Even though this previous reference study^28^ has a coarse spatial scale and was designed to run with global data to identify hotspots and regional trends of wetland vulnerability to sea-level rise, it constitutes a valuable initial point for comparison, as it can be used to estimate future changes in the estuary where our site is located. In fact, the data of surface elevation change rates from our site are part of the meta data used to compute the eco-geomorphic feedbacks in this vulnerability study^28^.

We also consider a variable rate of surface elevation change explicitly incorporating sediment-vegetation feedbacks into future surface elevation change rates, as considered in recent reviews^7^,^12^. We adapt the Kirwan model^12^, which is a modification of the Morris model^24^ for US saltmarshes, to our site. The rate of surface elevation change d*E/*d*t* at each grid point within the wetland and at each time step is computed using the following two equations:


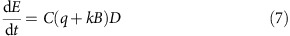



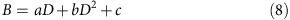


where *C* is the suspended sediment concentration in the water column, *B* is the vegetation aboveground biomass production, *D* is the depth below mean high tide and *q, k, a, b* and *c* are site-specific vegetation and depositional parameters. We obtain our own set of parameters for saltmarsh and mangrove based on local information including 10-year records from SET^43^, biomass measurements^56^ and suspended sediment concentrations^46^ (Table 1). *D* and *B* vary spatially and also temporally because they are updated considering changes in surface elevation and sea-level rise. We consider *C* either constant within each vegetation type or decreasing with distance to the main inlet at Fish Fry Creek (Supplementary Fig. 12). The variable rate of surface elevation change formulation is combined with the accelerated rate of sea-level change.

### Variable surface elevation change model parameters

Equations (7) and (8) have been originally developed for US saltmarshes and contain a number of vegetation and depositional parameters that are site-specific. We use our own local data to obtain the corresponding values of these parameters that best represent the conditions of the saltmarsh and mangroves in our site. Parameters *q* and *k* can be adjusted to represent different depositional characFteristics of wetlands, with *q* accounting for depositional processes exclusively due to sediment loading and *k* including the effectiveness of vegetation at capturing sediment and also organic matter accumulation in the soil and subsidence. For example, values of *q*=9 × 10^−5^ m^3^ yr^−1^ g^−1^ and *k*=7.5 × 10^−7^ m^5^ g^−2^ have been adopted based on observed surface elevation changes in the North Inlet, South Carolina, which is a *Spartina alterniflora* saltmarsh^12^,^24^ with tidal conditions similar to the ones at our site. Since *q* does not depend on vegetation, we adopt the same value of *q*=9 × 10^−5^ m^3^ yr^−1^ g^−1^ used for the North Inlet^12^,^24^ and we calibrate the value of *k* for saltmarsh and mangrove to match our local 10-year surface elevation data trends obtained using SET^43^ (Table 1). The calibration requires *C*, *B* and *D* values typical of our site. We adopt *C* values of 15 g m^−3^ in saltmarsh and 22 g m^−3^ in mangrove based on estimates for our site obtained from gravimetric analysis of grab samples^46^. In the absence of *B* measurements at the site we rely on measurements at a nearby wetland with similar tidal characteristics, where biomass values of 900 and 1000, g m^−2^ have been reported for the same saltmarsh species and similar size *A. marina* mangrove, respectively^56^. These biomass values can be used in equation (7) as proxy for biomass production^12^,^24^. Finally, typical *D* values of 0.142 and 0.474 m are selected for saltmarsh and mangrove, respectively, computed by averaging the *D* values predicted by our one-year simulation (Fig. 2h) over the corresponding vegetation type. Our resulting *k* values of 6.2 × 10^−7^ m^5^ g^−2^ for saltmarsh and 1.2 × 10^−7^ m^5^ g^−2^ for mangrove, are slightly lower but very close the values reported for US saltmarshes^12^,^24^. The lower *k* value for mangrove can be attributed to the fact that only a portion of the mangrove biomass is effective at trapping sediments.

The biomass coefficients *a, b* and *c* determine the distribution of biomass at different *D* values. The North Inlet saltmarshes have been described using a parabolic distribution with *a*=15,500, *b*=−18,550 and *c*=−1,364 (*D* in m and biomass in g m^−2^), which produces a curve that increases with *D* up to a maximum value of 1,868 g m^−2^ at *D*=0.4 m (coinciding with saltmarsh height) and then starts to decrease due to hypoxia^24^. Using the same rational for our shorter saltmarsh, we develop a parabolic distribution with a maximum biomass value of 1,050 g m^−2^ at *D*=0.25 m (coinciding with saltmarsh height). The maximum biomass value is obtained by extrapolating the value of 900 g m^−2^ at *D*=0.142 m mentioned before and considered typical of the conditions at the start of the long-term simulations. Our saltmarsh biomass curve coefficients are *a*=8,384, *b*=−16,767 and *c*=0.

In the absence of biomass curves for mangrove, we follow the same approach used in the Venice Lagoon^13^ by adapting the saltmarsh biomass curve structure to other vegetation types. We shift the range of *D* values for mangrove between 0.2 m (too shallow and saline for propagule establishment) and 1.1 m (too close to tidal channels), which match our observations in the wetland and are also consistent with our vegetation rules based on hydroperiod. Our mangrove biomass curve has a maximum of 1,225 g m^−2^ at *D*=0.65 m obtained by extrapolating the value of 1,000 g m^−2^ at *D*=0.474 m mentioned before and considered typical of the conditions at the start of the long-term simulations. Our mangrove biomass curve coefficients are *a*=15,698, *b*=−12,075 and *c*=−2,657.

### Spatially variable sediment concentration model

Spatially distributed models of wetland evolution that consider a variable surface elevation change formulation similar to equation (7) and (8) include a suspended sediment concentration value *C* that is either constant over the entire wetland (ref. 14) or decreases with increasing distance from tidal channels and from the wetland inlet (refs 13, 57, 58). Our results in Figs 4 and 5 can be considered to fall in between the two cases, as we use a constant *C* in mangrove and saltmarsh but the *C* values for mangroves (22 and 66 g m^−3^ for low and high sediment conditions, respectively) are higher than those for saltmarsh (15 and 45 g m^−3^ for low and high sediment conditions, respectively), and mangroves are typically closer to the wetland inlet and creeks than saltmarsh. To investigate whether a more detailed consideration of spatial variations in sediment concentration could affect our results, we developed a spatially variable sediment concentration model based on measurements in our site^46^, which includes three mangrove sites and six saltmarsh sites (Table 1).

Analysis of the data from the site reveals an exponential decay of the values of *C* with distance to the wetland inlet (Supplementary Fig. 12a), although not as strong as reported decays in other systems with a well-defined tidal network and no tidal restrictions (refs 57, 58). The values of *C* also increase linearly with measured values of *D* (Supplementary Fig. 12b). Because of the complex flow paths within the wetland implementing a model based on distance to the inlet of all 13,546 cells was impractical, so the empirical relationship between *C* and *D* was used to consider a variable sediment concentration in the model:


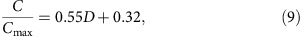


where *C*_max_ is the maximum value of concentration at the wetland inlet. We use *C*_max_=37 g m^−3^ to represent the existing low sediment conditions of the wetland, which results in 15 g m^–3^ at the average *D* of saltmarsh and 22 g m^−3^ at the average *D* of mangroves at the start of the long-term simulations, consistent with the constant sediment concentration computations of Fig. 4. To simulate high sediment conditions in the wetland corresponding to the constant sediment concentrations simulations of Fig. 5, we increase our *C*_max_ by a factor of three. Results of the variable sediment transport model incorporated into the bathtub and hydrodynamic approaches for low and high sediment concentrations are presented in Supplementary Figs 8–11.

### Field data for model set-up and parameterization

The field data used in this study for initial model set-up, calibration, validation and prediction was collected predominantly over the period 2004–2008 (refs 38, 39) (Table 1). It consists of the general landform of the study area, critical hydraulic controls acquired by real-time kinematic (RTK) global positioning system (GPS) (accurate to ±10 mm horizontal and ±20 mm vertical); mapping of estuarine habitat distribution obtained by combining RTK GPS and ground-truthed high-resolution aerial photography; vegetation morphological characteristics obtained using repeated nested quadrats; surface water level recorded with Solinst MLT 3001 pressure transducers (to ±5 mm); flow resistance values over vegetated substrates obtained by simultaneous measurements of water surface slopes with a Sokkia SDL30 automatic level sensor (to ±1 mm) and velocity with acoustic Doppler velocimetry (ADV) (to ±2.5 mm s^−1^) (ref. 59); suspended sediment concentration from grab samples gravimetrically analysed^46^ and soil surface elevation change on vegetated substrates measured by Surface Elevation Tables (SET^28^,^42^) (to ±1.4 mm) for the extended period 2002–2012 (ref. 43). 2004 water-level records collected at 15-min intervals from a tidal gauge situated 2 km from the field site were also used in this study.

### Data availability

The data sets generated during and/or analysed during the current study are available from the corresponding author on reasonable request.

## Additional information

**How to cite this article:** Rodríguez, J. F. *et al*. Potential increase in coastal wetland vulnerability to sea-level rise suggested by considering hydrodynamic attenuation effects. *Nat. Commun.* **8,** 16094 doi: 10.1038/ncomms16094 (2017).

**Publisher’s note**: Springer Nature remains neutral with regard to jurisdictional claims in published maps and institutional affiliations.

## Supplementary Material

Supplementary Information

## Figures and Tables

**Table 1:** Field data.

**Variable**	**Value**	**Comments and references**
Mean estuary level 2004	0.07 mAHD	2004 tidal record at nearby station
Mean High Tide 2004	0.6 mAHD	2004 tidal record at nearby station
Saltmarsh elevation	>0.4 mAHD	N*=382 (ref. 38)
Mangrove elevation	<0.43 mAHD	*N*=600 (ref. 38)
Saltmarsh height	0.25 m	*N*=382 (ref. 38)
Mangrove pneumatophore height	0.14 m	*N*=600 (ref. 38)
Saltmarsh spring hydroperiod	<1	*N*=382 (ref. 38)
Mangrove spring hydroperiod	<0.45	*N*=600 (ref. 38)
Saltmarsh *D*	<0.3 m	*N*=382 (ref. 38)
Mangrove *D*	>0.29 m	*N*=600 (ref. 38)
Saltmarsh *n*	0.05–0.6	*N*=12 (ref. 59)
Mangrove pneumatophores *n*	0.05–0.35	*N*=11 (ref. 59)
Unvegetated *n*	0.02	*N*=15 (ref. 59)
Saltmarsh suspended sediment conc.	13–28 g m^−3^	*N*=6 (ref. 46)
Mangrove suspended sediment conc.	28–40 g m^−3^	*N*=3 (ref. 46)
Saltmarsh surface elevation change	1.39 mm yr^−1^	*N*=17 Ten-year trend 2002–2012 (ref. 43)
Mangrove surface elevation change	2.23 mm yr^−1^	*N*=17 Ten-year trend 2002–2012 (ref. 43)

mAHD stands for metres above the Australian height datum, which approximates mean sea level.

^*^N is the number of samples.
