# Supplementary material for: Potential increase in coastal wetland vulnerability to sea-level rise suggested by considering hydrodynamic attenuation effects
Source: Nat Commun. 2017 Jul 13;8:16094. doi: 10.1038/ncomms16094 (PMC5511368; doi:10.1038/ncomms16094)
Supplement: Supplementary Information [file ncomms16094-s1.pdf]

File name: Supplementary Information
Description: Supplementary Figures and Supplementary Tables

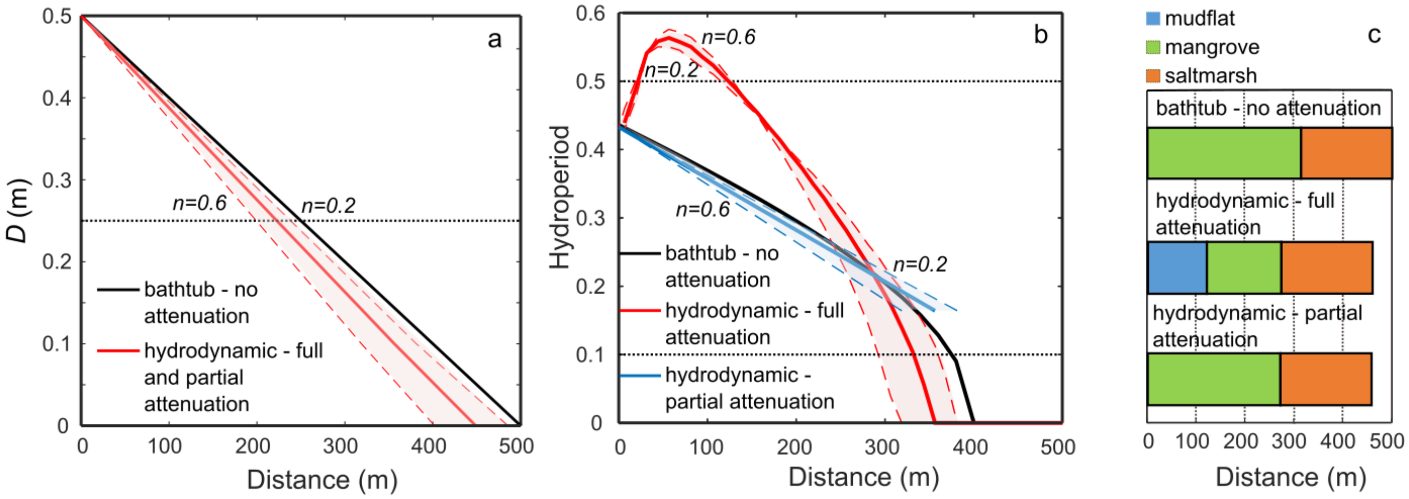

Supplementary Figure 1 | Vegetation prediction using full attenuation and simplified partial attenuation flow models. a, Changes in *D* obtained from both models are identical and show the effects of attenuation on *D* assuming a vegetative flow resistance that corresponds to saltmarsh and mangrove, represented by a range of Manning's *n* values from 0.2 to 0.6 (shaded areas) with a mean of 0.4 (solid line). **b**, Changes in hydroperiod computed at an offset of 14 cm along the tidal flat for both approaches indicate a significant impact of attenuation on the hydroperiod when full attenuation is considered, which is not captured by the simplified partial attenuation model. **c**, Vegetation distribution simulated using the two modelling approaches shows that the partial attenuation approach produces a distribution very close to the results using the bathtub model.

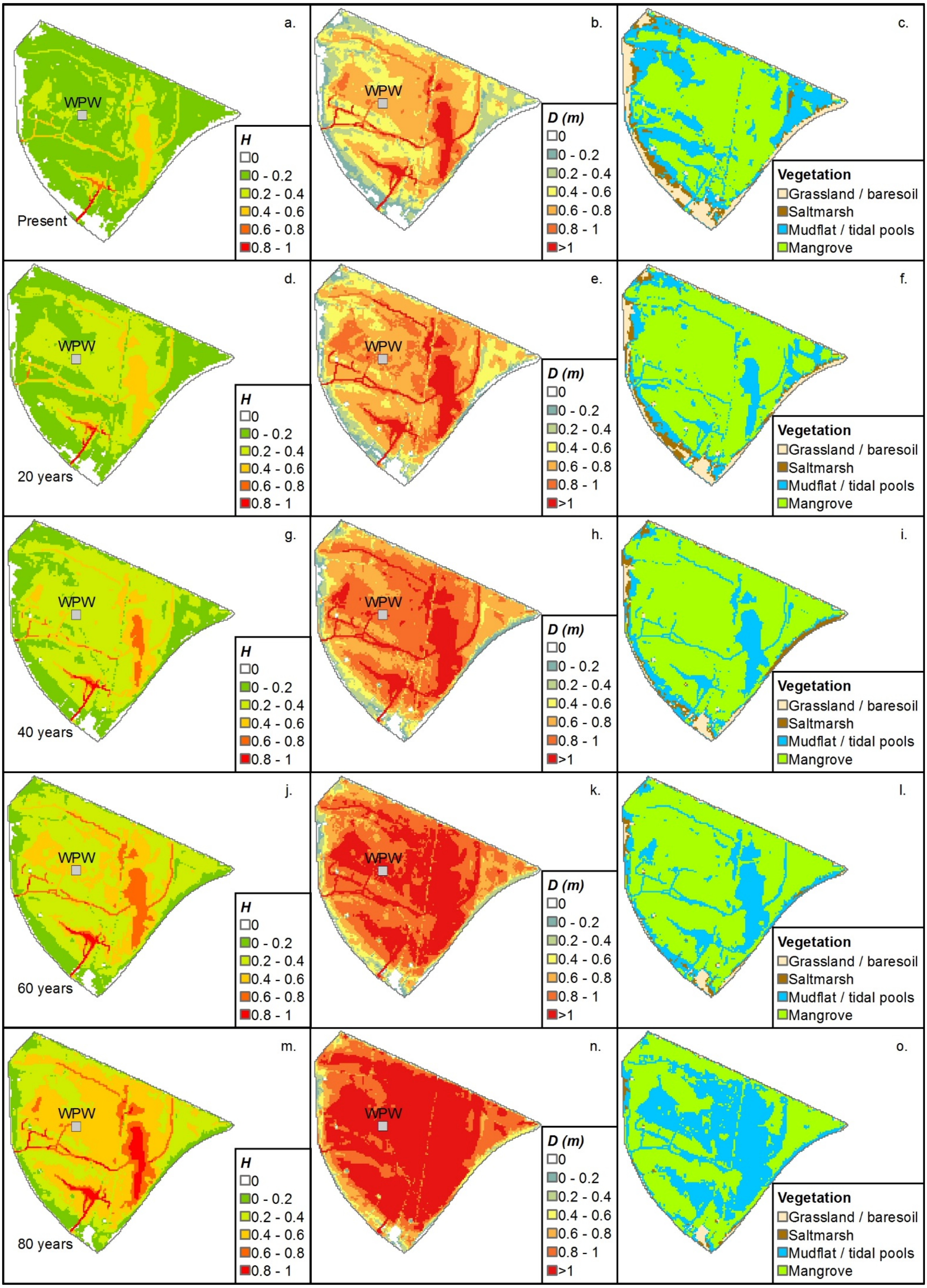

Supplementary Figure 2 | Long term wetland evolution for constant rate of sea-level rise and constant rate of soil surface elevation change using the “bathtub” approach without considering the effects of flow attenuation. *H* = hydroperiod, *D* = depth below mean high tide

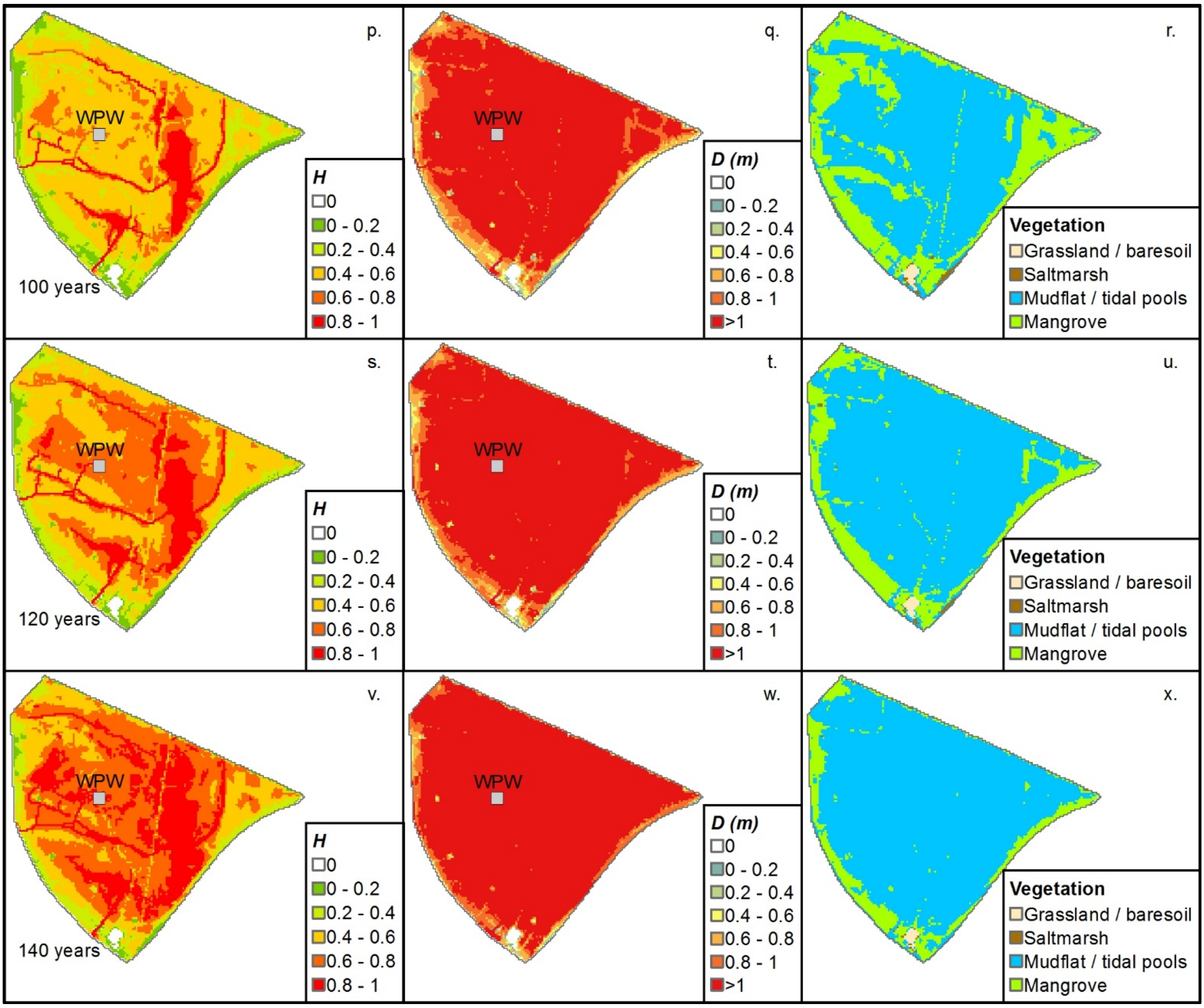

Supplementary Figure 2 (cont.)

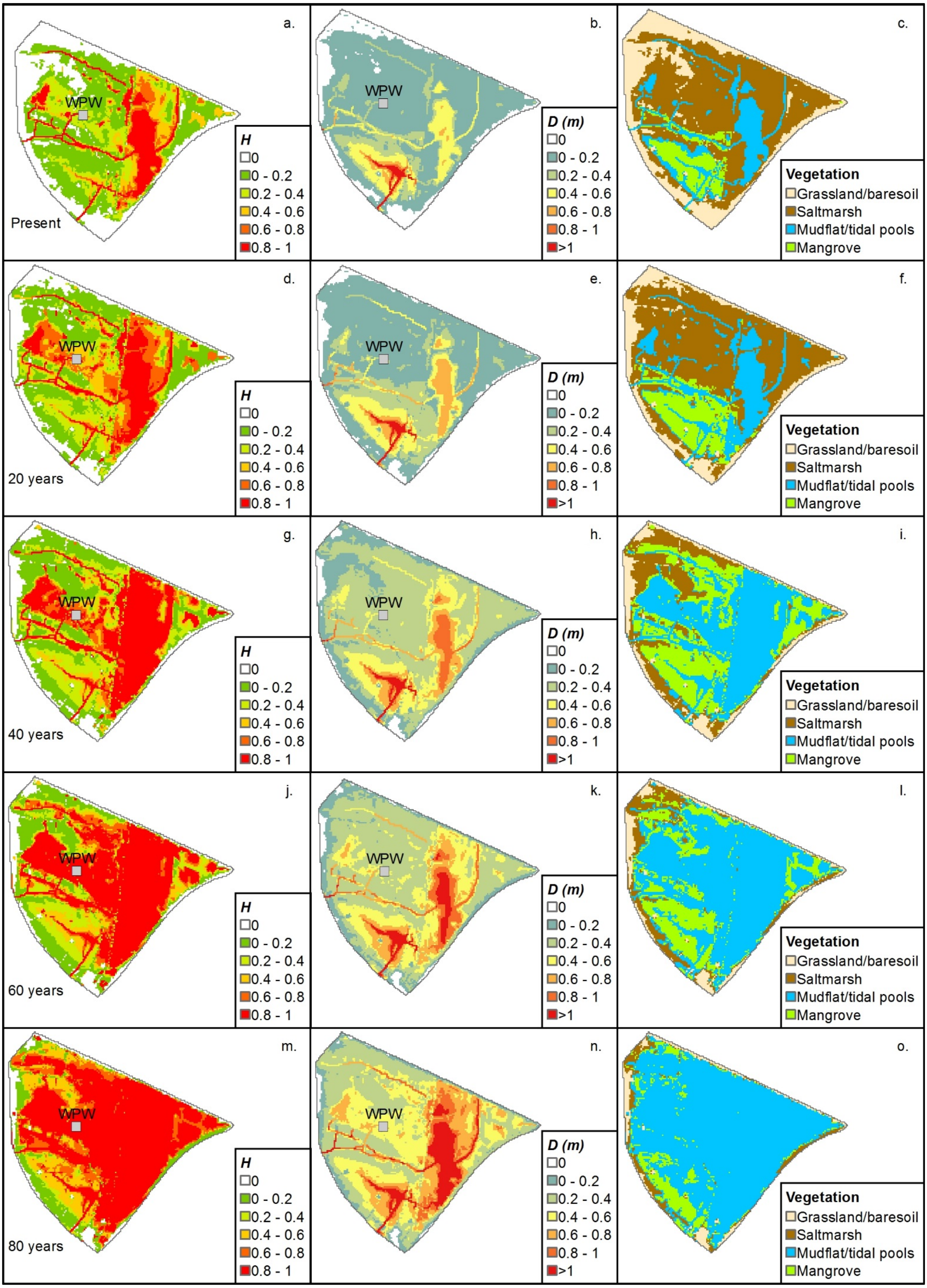

Supplementary Figure 3 | Long term wetland evolution for constant rate of sea-level rise and constant rate of soil surface elevation change using the hydrodynamic approach considering the full effects of flow attenuation. *H* = hydroperiod, *D* = depth below mean high tide.

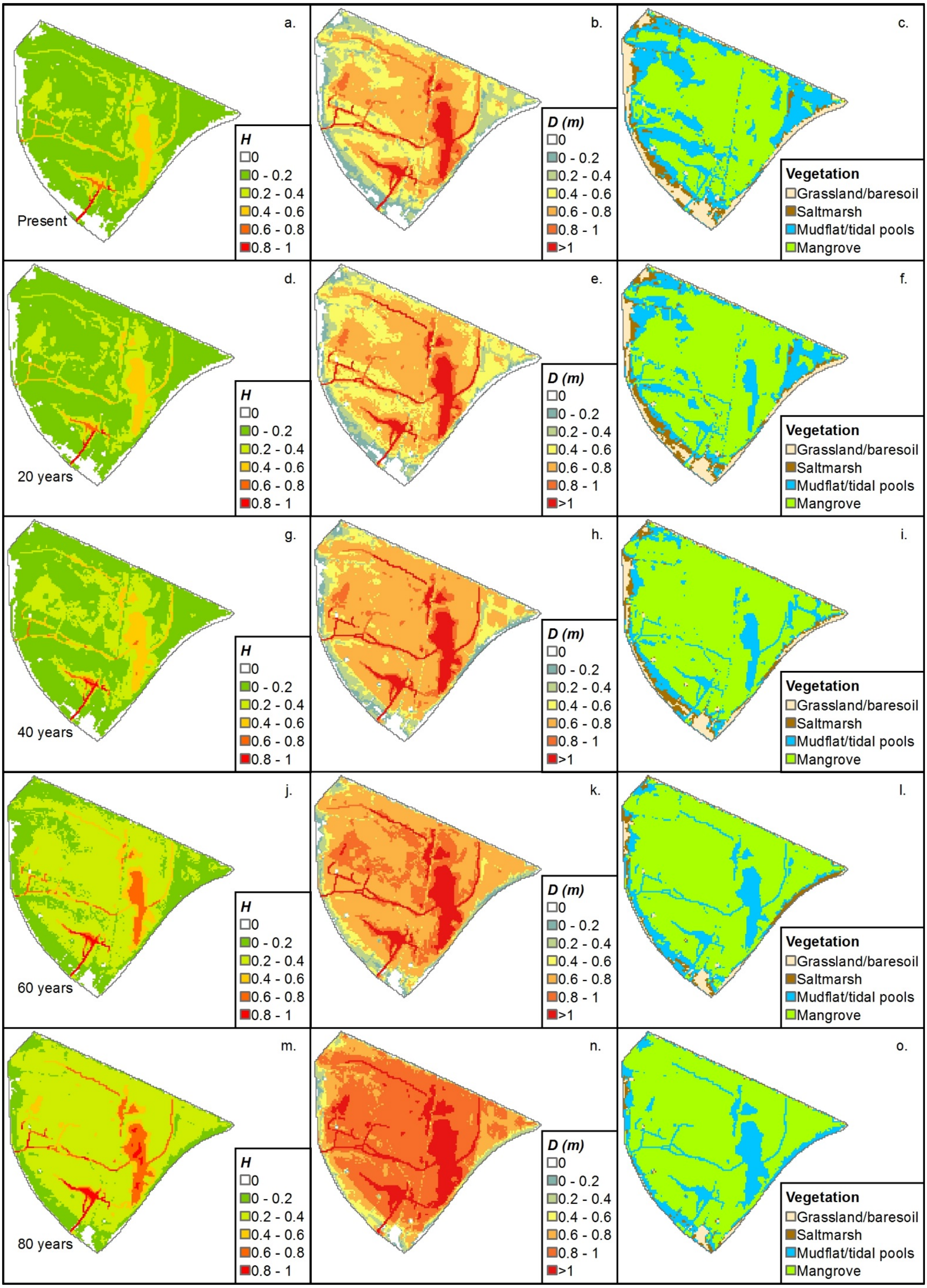

Supplementary Figure 4 | Long term wetland evolution for variable rate of sea-level rise and variable rate of soil surface elevation change with low sediment load using the "bathtub" approach without considering the effects of flow attenuation. *H* = hydroperiod, *D* = depth below mean high tide

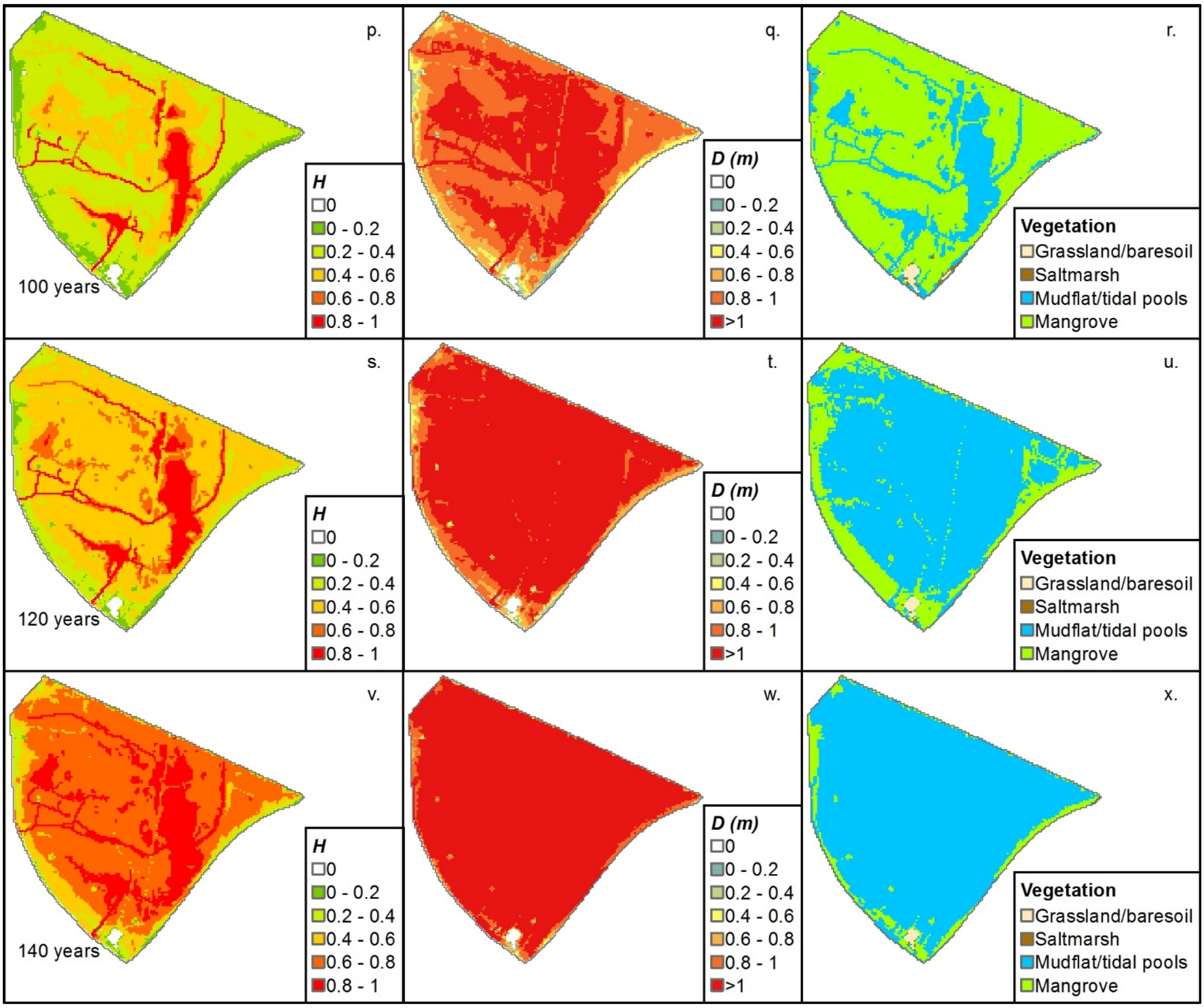

Supplementary Figure 4 (cont.)

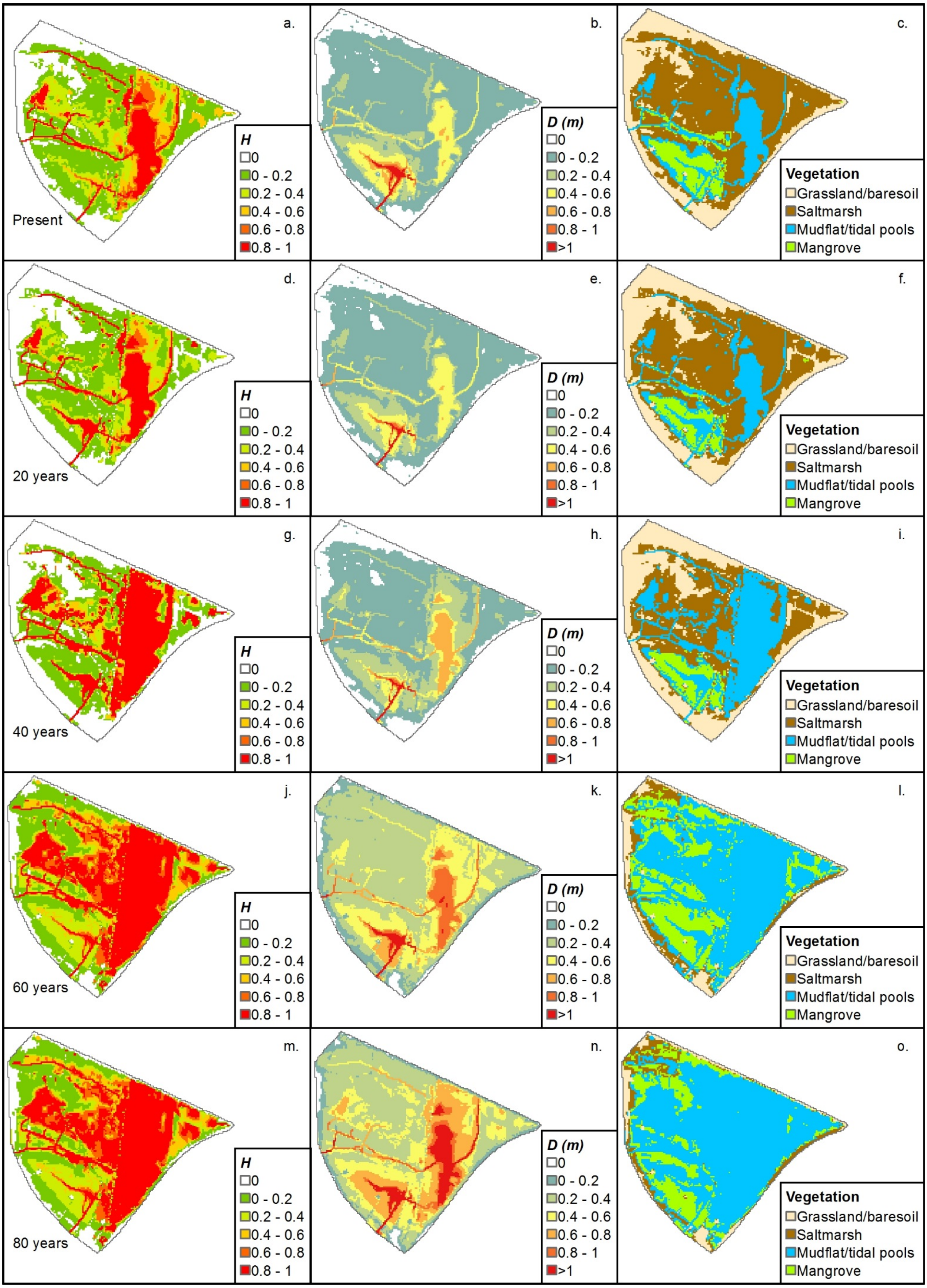

Supplementary Figure 5 | Long term wetland evolution for variable rate of sea-level rise and variable rate of soil surface elevation change with low sediment load using the hydrodynamic approach considering the full effects of flow attenuation. *H* = hydroperiod, *D* = depth below mean high tide.

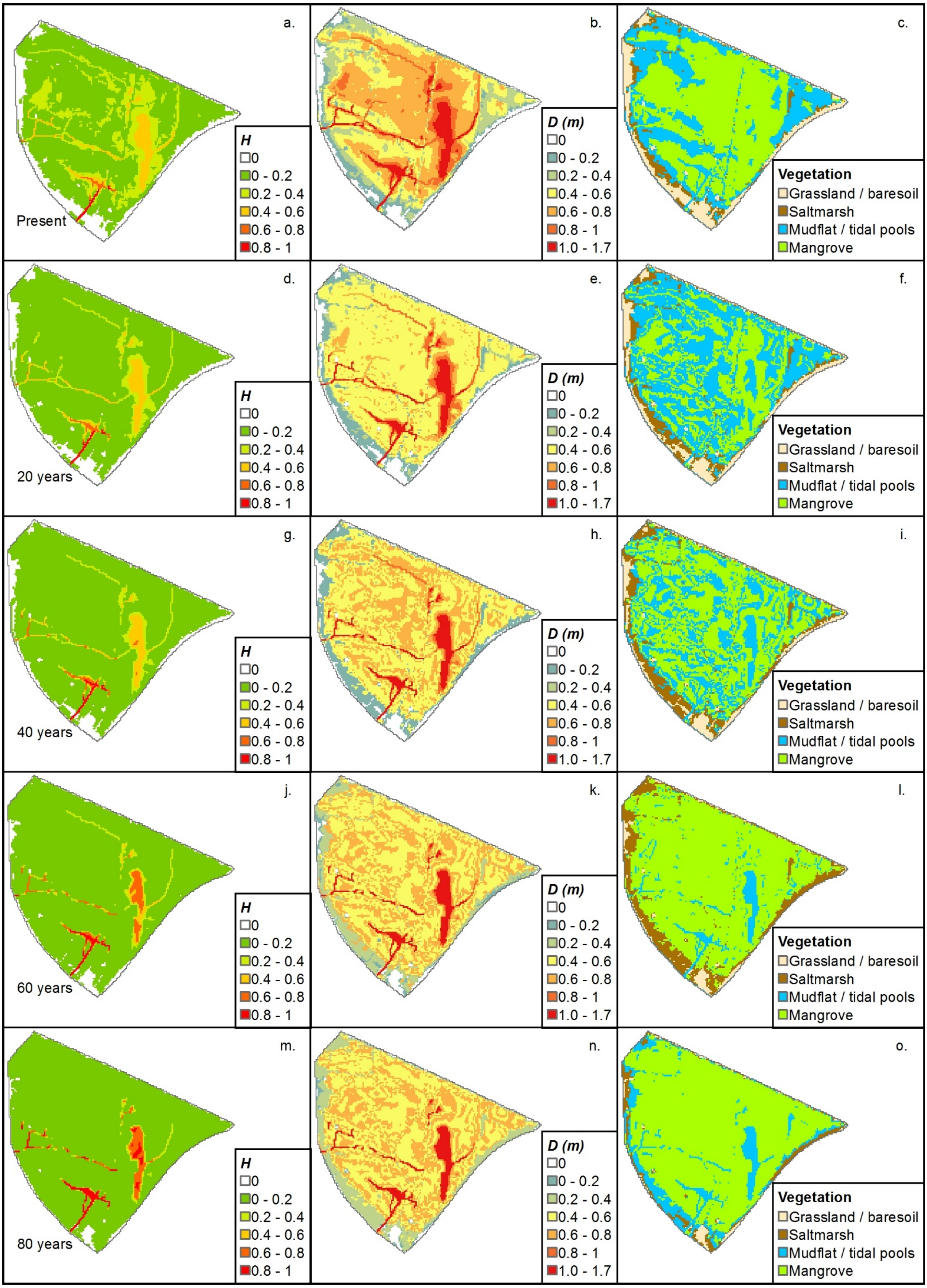

Supplementary Figure 6 | Long term wetland evolution for variable rate of sea-level rise and variable rate of soil surface elevation change with high sediment load using the "bathtub" approach without considering the effects of flow attenuation. *H* = hydroperiod, *D* = depth below mean high tide

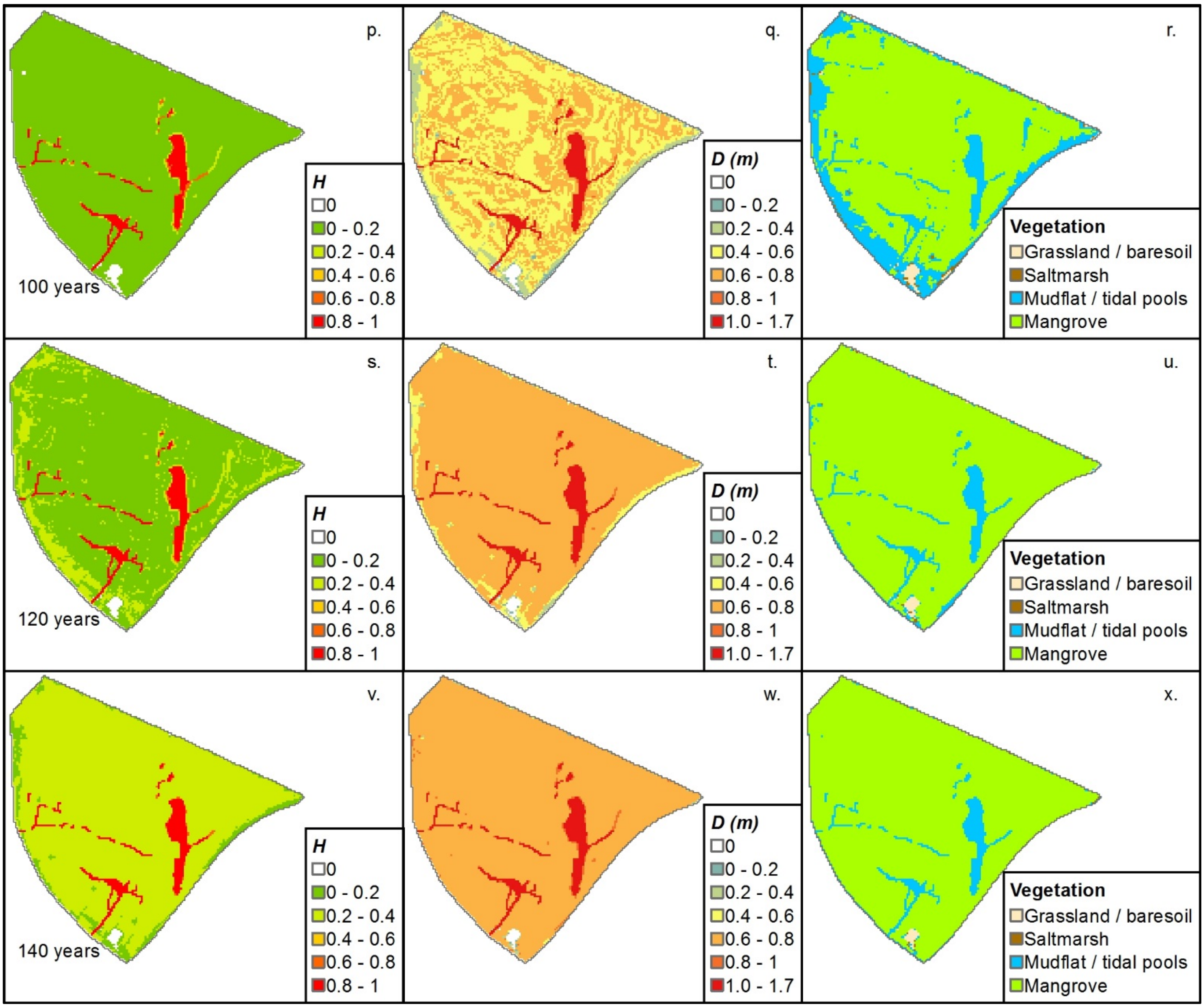

Supplementary Figure 6 (cont.)

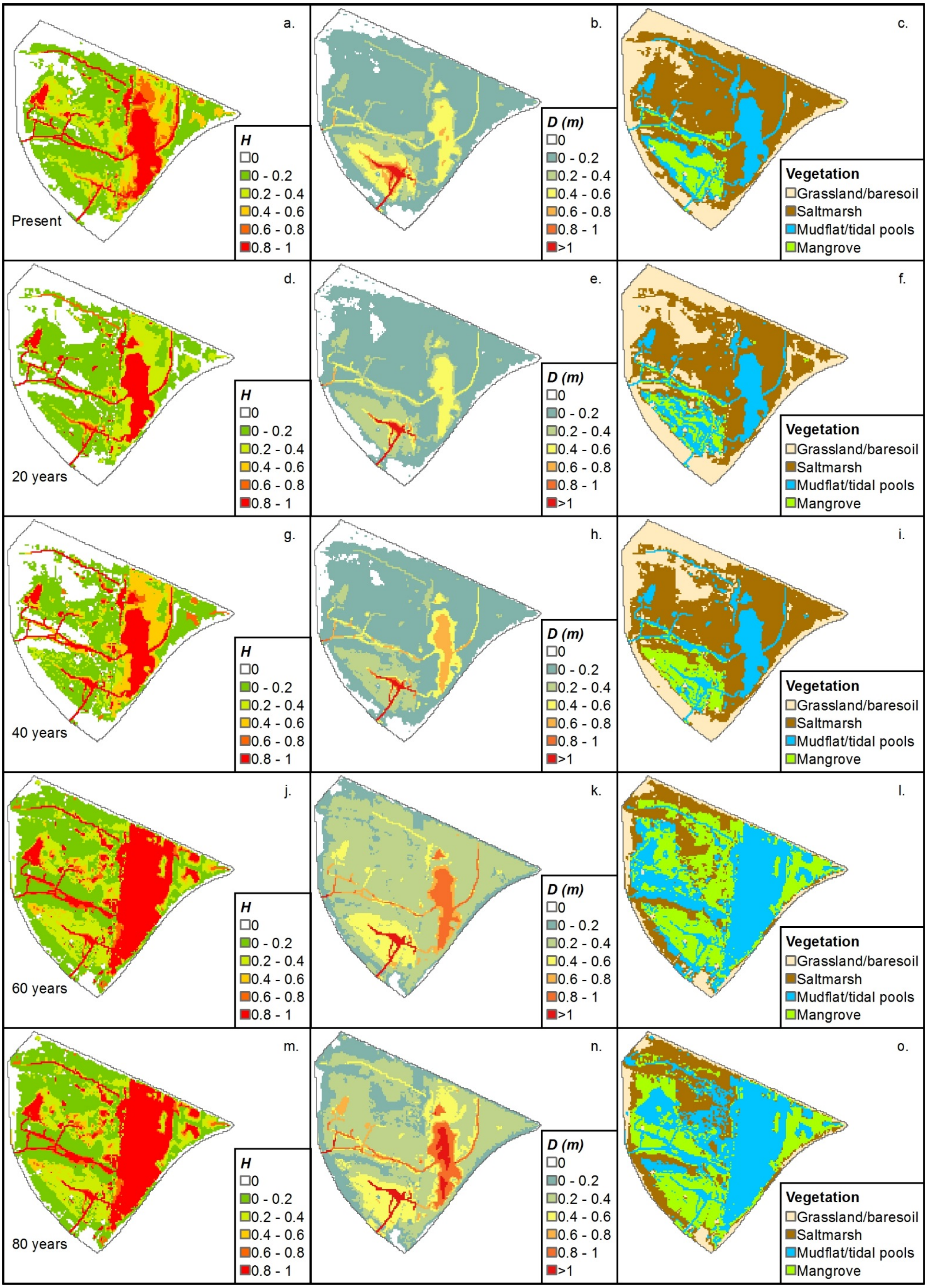

Supplementary Figure 7 | Long term wetland evolution for variable rate of sea-level rise and variable rate of soil surface elevation change with high sediment load using the hydrodynamic approach considering the full effects of flow attenuation. *H* = hydroperiod, *D* = depth below mean high tide.

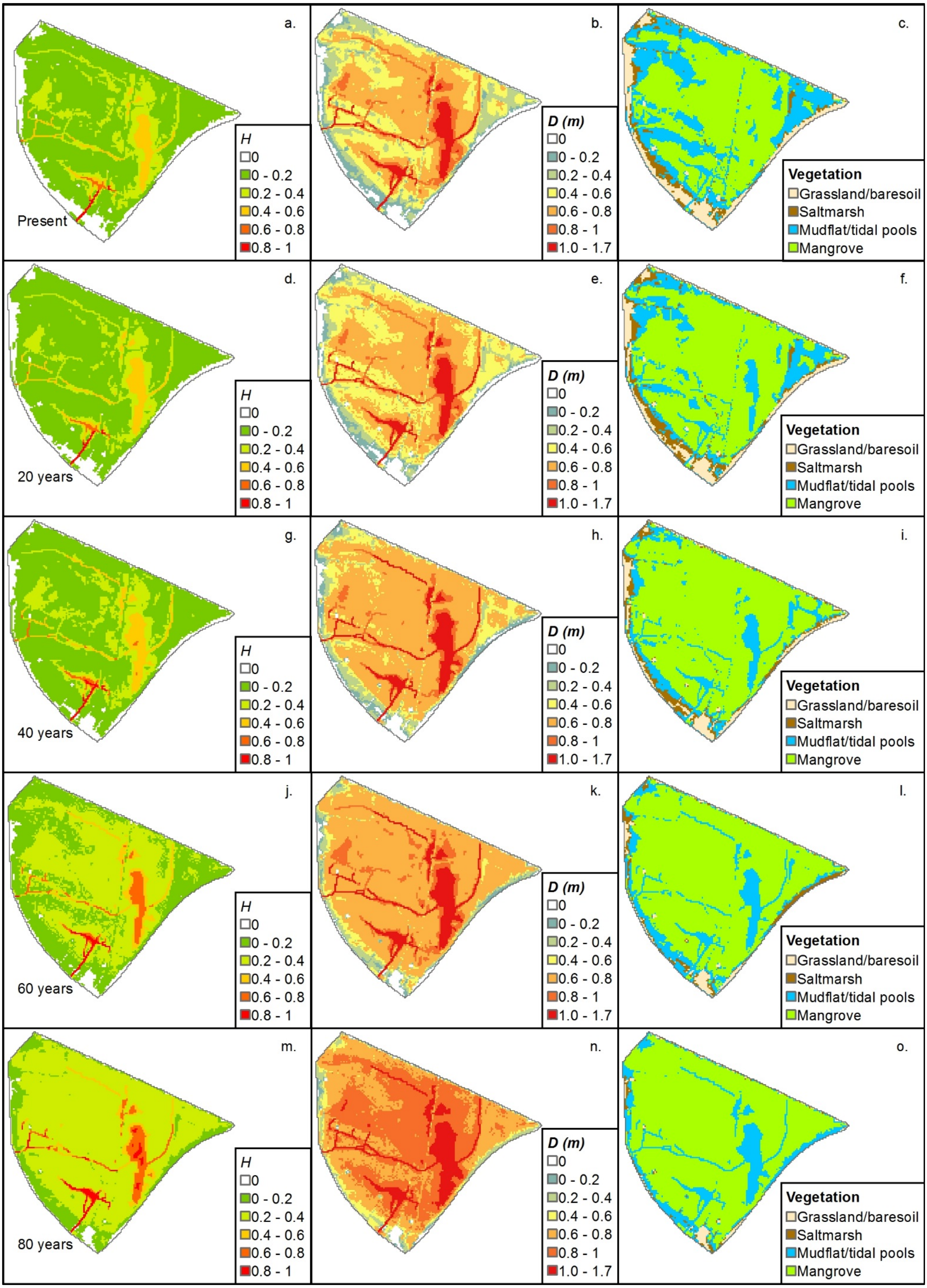

Supplementary Figure 8 | Long term wetland evolution for variable rate of sea-level rise and variable rate of soil surface elevation change and concentration with low sediment load using the "bathtub" approach without considering the effects of flow attenuation. *H* = hydroperiod, *D* = depth below mean high tide

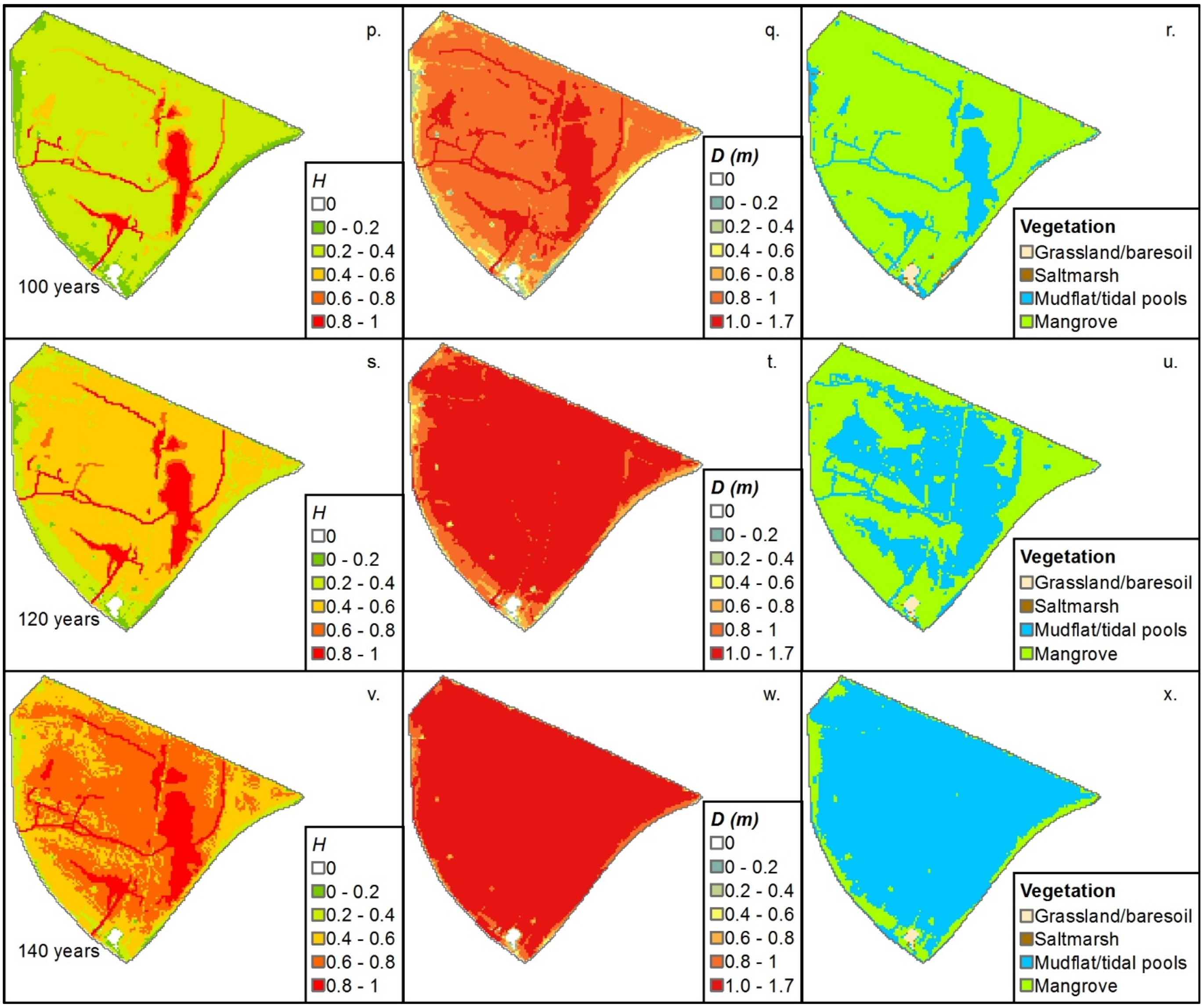

Supplementary Figure 8 (cont.)

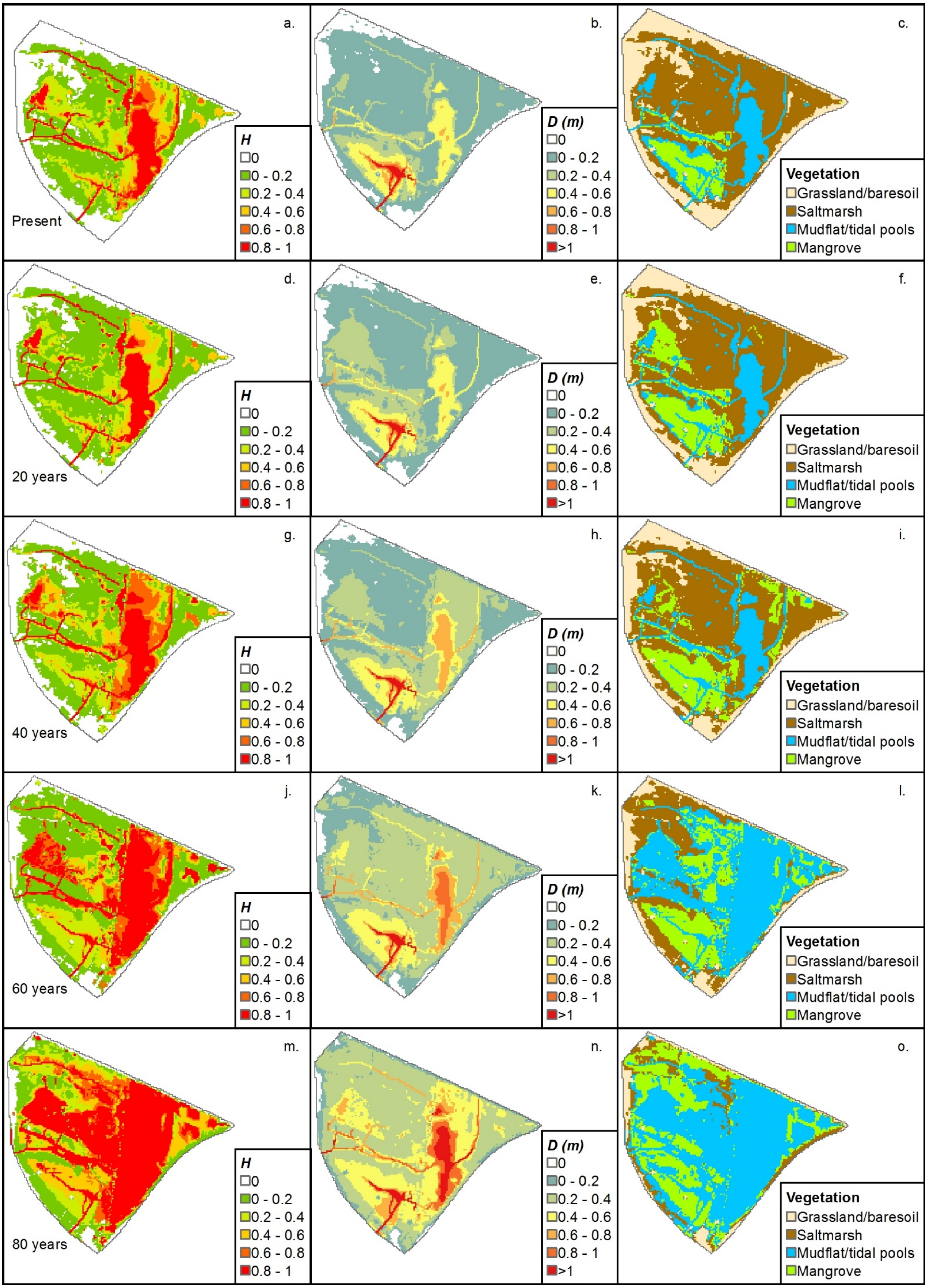

Supplementary Figure 9 | Long term wetland evolution for variable rate of sea-level rise and variable rate of soil surface elevation change and concentration with low sediment load using the hydrodynamic approach considering the full effects of flow attenuation. *H* = hydropeiod, *D* = depth below mean high tide.

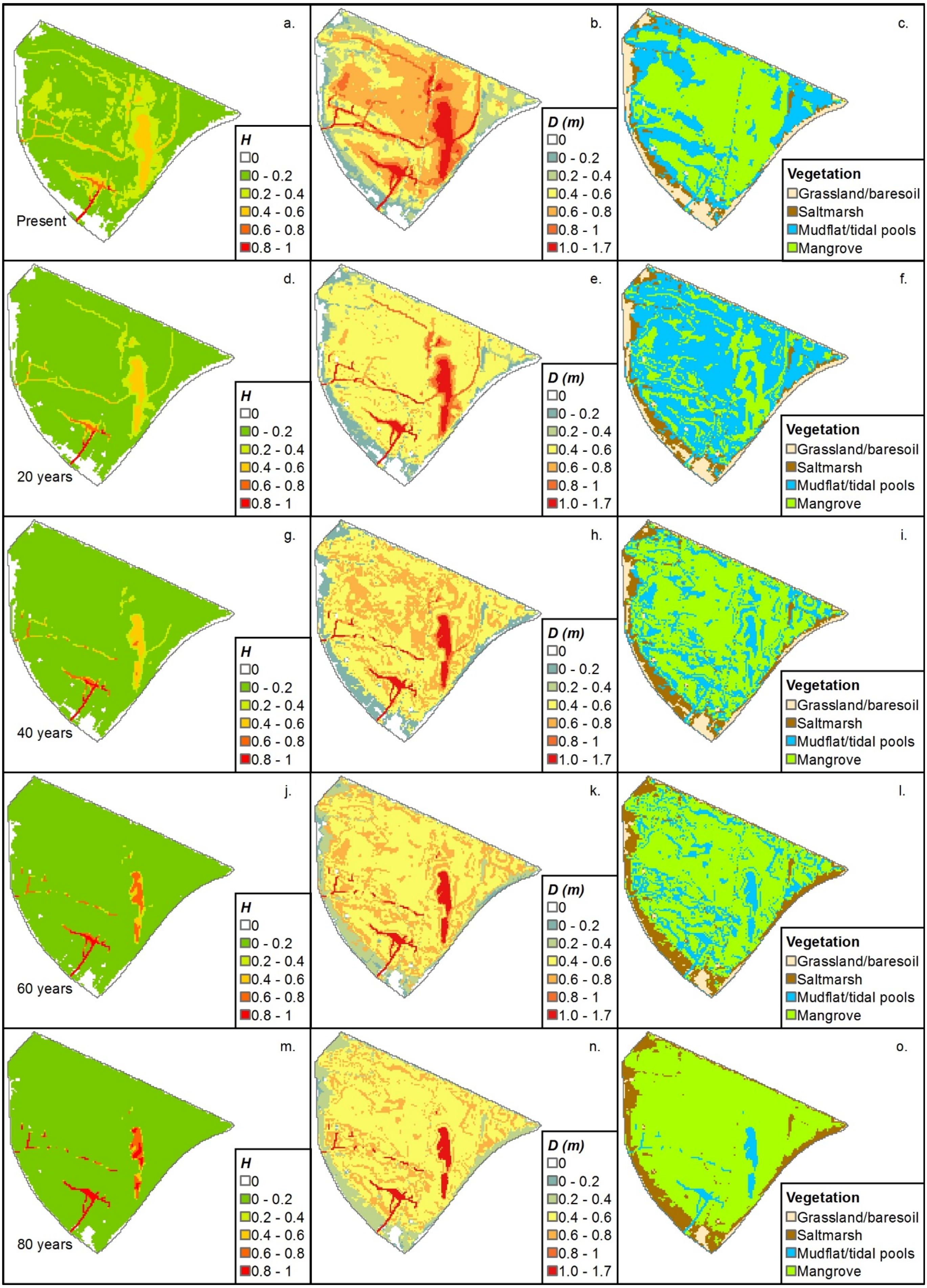

Supplementary Figure 10 | Long term wetland evolution for variable rate of sea-level rise and variable rate of soil surface elevation change and concentration with high sediment load using the "bathtub" approach without considering the effects of flow attenuation. *H* = hydroperiod, *D* = depth below mean high tide

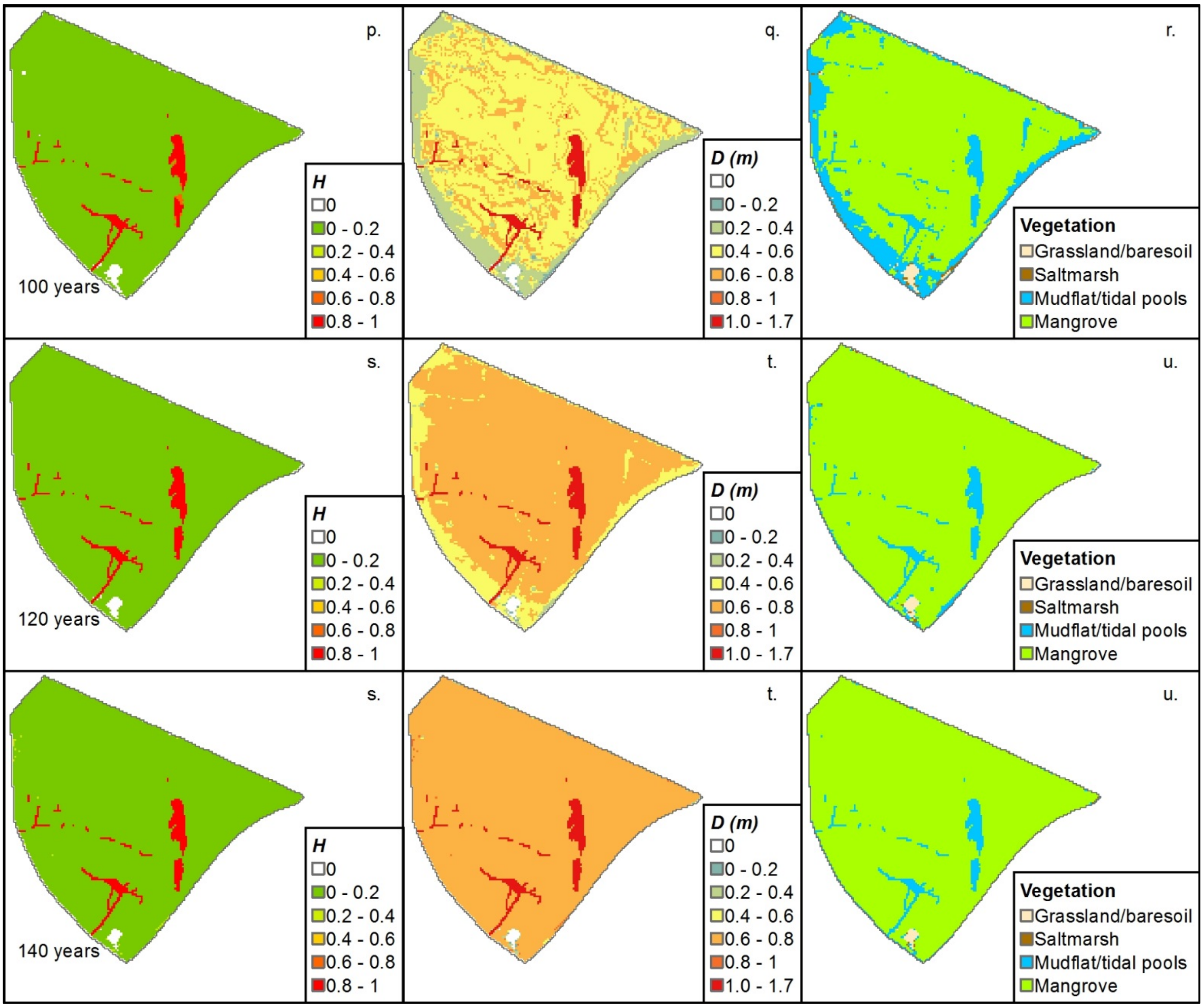

Supplementary Figure 10 (cont.)

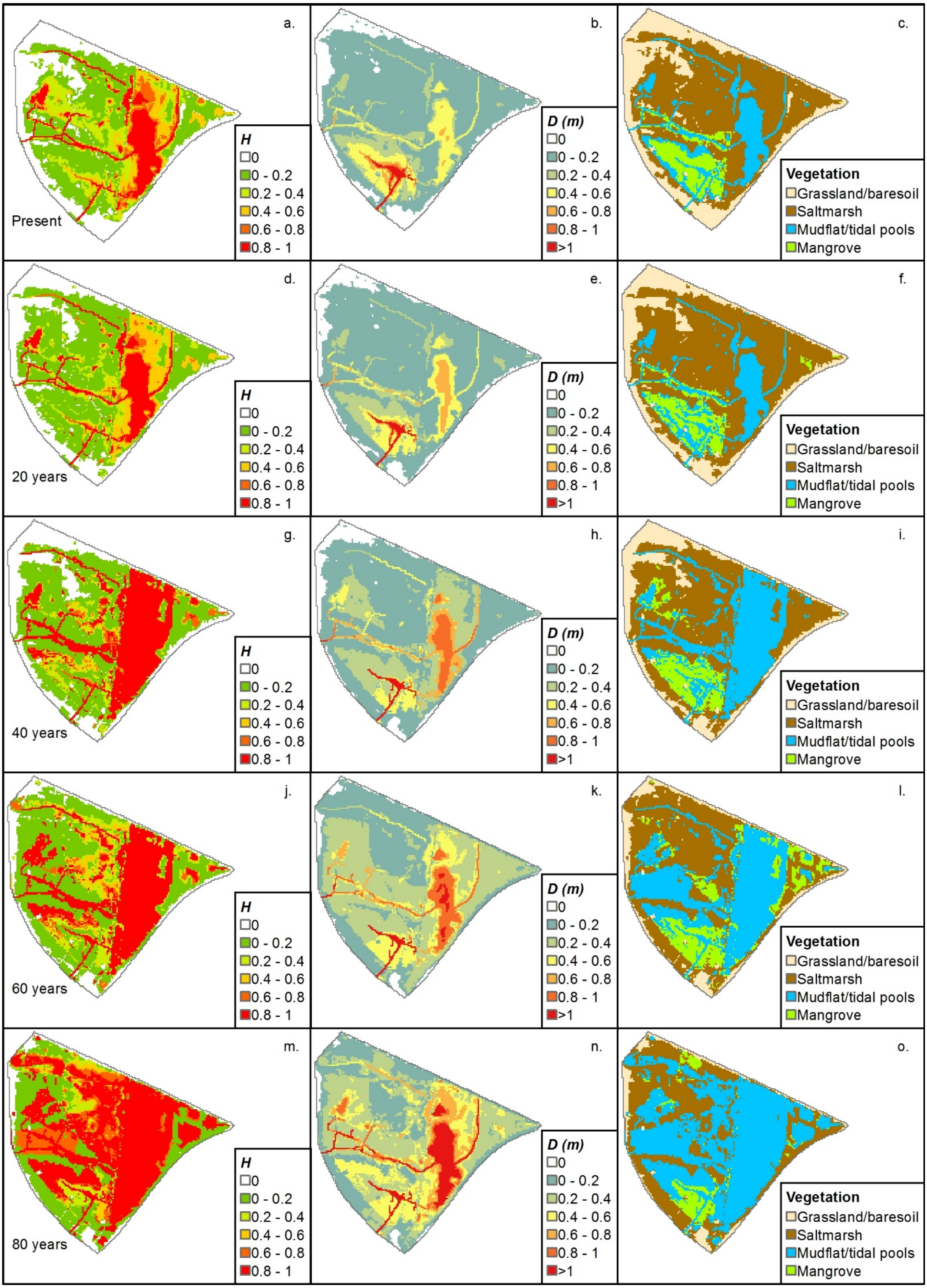

Supplementary Figure 11 | Long term wetland evolution for variable rate of sea-level rise and variable rate of soil surface elevation change and concentration with high sediment load using the hydrodynamic approach considering the full effects of flow attenuation. *H* = hydropeiod, *D* = depth below mean high tide.

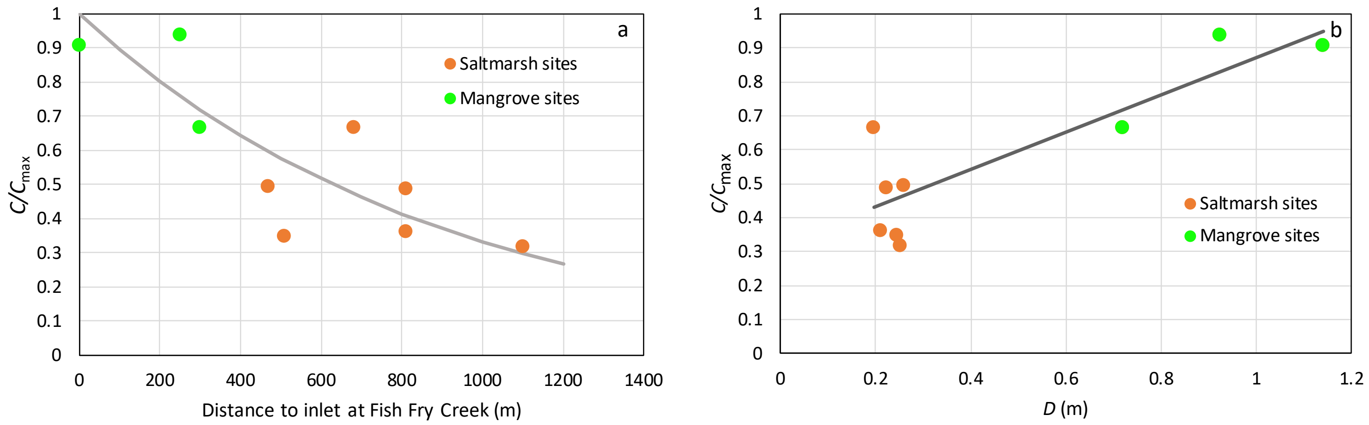

Supplementary Figure 12 | Variation of suspended sediment in the wetland. a, Values of suspended sediment concentration decay exponentially with distance to the inlet at Fish Fry Cheek, both in saltmarsh and mangrove sites. **b,** The same sites show a linear increase of concentration with depth below mean high tide *D*. Lines of best fit have been included in the figures.

Supplementary Table 1 | Data used during calibration and validation of the hydrodynamic model.

Series	Record period	Initial Date	Final Date
Calibration	888 hours	12/09/2004	20/10/2004
Validation	97 hours	31/12/2005	04/01/2006

Supplementary Table 2 | Manning roughness intervals tested.

Type of element / soil coverage	Roughness coefficient interval	Final calibrated values
Unvegetated	0.030 – 0.100	0.035
Mangrove (pneumatophores and stems)	0.100 – 0.700	0.500
Saltmarsh	0.100 – 0.500	0.150

Supplementary Table 3 | Performance indicators of the hydrodynamic model for calibration and validation.

Performance indicators	Calibration	Validation
r	0.6	0.8
RSR	0.1	0.7
PBIAS (%)	6.1	3.4
NSE	0.9	0.5
